# Dense sampling of bird diversity increases power of comparative genomics

**DOI:** 10.1038/s41586-020-2873-9

**Published:** 2020-11-11

**Authors:** Shaohong Feng, Josefin Stiller, Yuan Deng, Joel Armstrong, Qi Fang, Andrew Hart Reeve, Duo Xie, Guangji Chen, Chunxue Guo, Brant C. Faircloth, Bent Petersen, Zongji Wang, Qi Zhou, Mark Diekhans, Wanjun Chen, Sergio Andreu-Sánchez, Ashot Margaryan, Jason Travis Howard, Carole Parent, George Pacheco, Mikkel-Holger S. Sinding, Lara Puetz, Emily Cavill, Ângela M. Ribeiro, Leopold Eckhart, Jon Fjeldså, Peter A. Hosner, Robb T. Brumfield, Les Christidis, Mads F. Bertelsen, Thomas Sicheritz-Ponten, Dieter Thomas Tietze, Bruce C. Robertson, Gang Song, Gerald Borgia, Santiago Claramunt, Irby J. Lovette, Saul J. Cowen, Peter Njoroge, John Philip Dumbacher, Oliver A. Ryder, Jérôme Fuchs, Michael Bunce, David W. Burt, Joel Cracraft, Guanliang Meng, Shannon J. Hackett, Peter G. Ryan, Knud Andreas Jønsson, Ian G. Jamieson, Rute R. da Fonseca, Edward L. Braun, Peter Houde, Siavash Mirarab, Alexander Suh, Bengt Hansson, Suvi Ponnikas, Hanna Sigeman, Martin Stervander, Paul B. Frandsen, Henriette van der Zwan, Rencia van der Sluis, Carina Visser, Christopher N. Balakrishnan, Andrew G. Clark, John W. Fitzpatrick, Reed Bowman, Nancy Chen, Alison Cloutier, Timothy B. Sackton, Scott V. Edwards, Dustin J. Foote, Subir B. Shakya, Frederick H. Sheldon, Alain Vignal, André E. R. Soares, Beth Shapiro, Jacob González-Solís, Joan Ferrer-Obiol, Julio Rozas, Marta Riutort, Anna Tigano, Vicki Friesen, Love Dalén, Araxi O. Urrutia, Tamás Székely, Yang Liu, Michael G. Campana, André Corvelo, Robert C. Fleischer, Kim M. Rutherford, Neil J. Gemmell, Nicolas Dussex, Henrik Mouritsen, Nadine Thiele, Kira Delmore, Miriam Liedvogel, Andre Franke, Marc P. Hoeppner, Oliver Krone, Adam M. Fudickar, Borja Milá, Ellen D. Ketterson, Andrew Eric Fidler, Guillermo Friis, Ángela M. Parody-Merino, Phil F. Battley, Murray P. Cox, Nicholas Costa Barroso Lima, Francisco Prosdocimi, Thomas Lee Parchman, Barney A. Schlinger, Bette A. Loiselle, John G. Blake, Haw Chuan Lim, Lainy B. Day, Matthew J. Fuxjager, Maude W. Baldwin, Michael J. Braun, Morgan Wirthlin, Rebecca B. Dikow, T. Brandt Ryder, Glauco Camenisch, Lukas F. Keller, Jeffrey M. DaCosta, Mark E. Hauber, Matthew I. M. Louder, Christopher C. Witt, Jimmy A. McGuire, Joann Mudge, Libby C. Megna, Matthew D. Carling, Biao Wang, Scott A. Taylor, Glaucia Del-Rio, Alexandre Aleixo, Ana Tereza Ribeiro Vasconcelos, Claudio V. Mello, Jason T. Weir, David Haussler, Qiye Li, Huanming Yang, Jian Wang, Fumin Lei, Carsten Rahbek, M. Thomas P. Gilbert, Gary R. Graves, Erich D. Jarvis, Benedict Paten, Guojie Zhang

**Affiliations:** 1 China National GeneBank, BGI-Shenzhen, Shenzhen, China; 2 State Key Laboratory of Genetic Resources and Evolution, Kunming Institute of Zoology, Chinese Academy of Sciences, Kunming, China; 3 BGI-Shenzhen, Shenzhen, China; 4 Villum Centre for Biodiversity Genomics, Section for Ecology and Evolution, Department of Biology, University of Copenhagen, Copenhagen, Denmark; 5 UC Santa Cruz Genomics Institute, UC Santa Cruz, Santa Cruz, CA USA; 6 Natural History Museum of Denmark, University of Copenhagen, Copenhagen, Denmark; 7 BGI Education Center, University of Chinese Academy of Sciences, Shenzhen, China; 8 Department of Biological Sciences, Louisiana State University, Baton Rouge, LA USA; 9 Museum of Natural Science, Louisiana State University, Baton Rouge, LA USA; 10 Centre of Excellence for Omics-Driven Computational Biodiscovery (COMBio), Faculty of Applied Sciences, AIMST University, Kedah, Malaysia; 11 Section for Evolutionary Genomics, The GLOBE Institute, Faculty of Health and Medical Sciences, University of Copenhagen, Copenhagen, Denmark; 12 MOE Laboratory of Biosystems Homeostasis and Protection, Life Sciences Institute, Zhejiang University, Hangzhou, China; 13 Department of Neuroscience and Developmental Biology, University of Vienna, Vienna, Austria; 14 Center for Reproductive Medicine, The 2nd Affiliated Hospital, School of Medicine, Zhejiang University, Hangzhou, China; 15 Institute of Molecular Biology, National Academy of Sciences, Yerevan, Armenia; 16 Novogene, Durham, NC USA; 17 Duke University Medical Center, Durham, NC USA; 18 Department of Dermatology, Medical University of Vienna, Vienna, Austria; 19 Center for Macroecology, Evolution, and Climate, GLOBE Institute, University of Copenhagen, Copenhagen, Denmark; 20 Southern Cross University, Coffs Harbour, New South Wales Australia; 21 Centre for Zoo and Wild Animal Health, Copenhagen Zoo, Frederiksberg, Denmark; 22 Center of Natural History, Universität Hamburg, Hamburg, Germany; 23 Department of Zoology, University of Otago, Dunedin, New Zealand; 24 Key Laboratory of Zoological Systematics and Evolution, Institute of Zoology, Chinese Academy of Sciences, Beijing, China; 25 Environmental Futures Research Institute, Griffith University, Nathan, Queensland Australia; 26 Department of Biology, University of Maryland, College Park, MD USA; 27 Department of Natural History, Royal Ontario Museum, Toronto, Ontario Canada; 28 Department of Ecology and Evolutionary Biology, University of Toronto, Toronto, Ontario Canada; 29 Cornell Lab of Ornithology, Cornell University, Ithaca, NY USA; 30 Biodiversity and Conservation Science, Department of Biodiversity Conservation and Attractions, Perth, Western Australia Australia; 31 Ornithology Section, Zoology Department, National Museums of Kenya, Nairobi, Kenya; 32 Ornithology and Mammalogy, California Academy of Sciences, San Francisco, CA USA; 33 San Diego Zoo Institute for Conservation Research, Escondido, CA USA; 34 Evolution, Behavior, and Ecology, Division of Biology, University of California San Diego, La Jolla, CA USA; 35 Institut de Systématique, Evolution, Biodiversité (ISYEB), Muséum National d’Histoire Naturelle, CNRS, Sorbonne Université, EPHE, Université des Antilles, Paris, France; 36 Trace and Environmental DNA (TrEnD) Laboratory, School of Molecular and Life Sciences, Curtin University, Western Australia Perth, Australia; 37 UQ Genomics, University of Queensland, Brisbane, Queensland Australia; 38 Department of Ornithology, American Museum of Natural History, New York, NY USA; 39 Integrative Research Center, Field Museum of Natural History, Chicago, IL USA; 40 FitzPatrick Institute of African Ornithology, University of Cape Town, Cape Town, South Africa; 41 Department of Biology, University of Florida, Gainesville, FL USA; 42 Department of Biology, New Mexico State University, Las Cruces, NM USA; 43 Department of Electrical and Computer Engineering, University of California San Diego, La Jolla, CA USA; 44 Department of Ecology and Genetics – Evolutionary Biology, Evolutionary Biology Centre (EBC), Science for Life Laboratory, Uppsala University, Uppsala, Sweden; 45 Department of Organismal Biology – Systematic Biology, Evolutionary Biology Centre (EBC), Science for Life Laboratory, Uppsala University, Uppsala, Sweden; 46 School of Biological Sciences, University of East Anglia, Norwich, UK; 47 Department of Biology, Lund University, Lund, Sweden; 48 Institute of Ecology and Evolution, University of Oregon, Eugene, OR USA; 49 Department of Plant and Wildlife Sciences, Brigham Young University, Provo, UT USA; 50 Data Science Lab, Office of the Chief Information Officer, Smithsonian Institution, Washington, DC USA; 51 Focus Area for Human Metabolomics, North-West University, Potchefstroom, South Africa; 52 Department of Animal Sciences, University of Pretoria, Pretoria, South Africa; 53 Department of Biology, East Carolina University, Greenville, NC USA; 54 Department of Molecular Biology and Genetics, Cornell University, Ithaca, NY USA; 55 Avian Ecology Program, Archbold Biological Station, Venus, FL USA; 56 Department of Biology, University of Rochester, Rochester, NY USA; 57 Department of Organismic and Evolutionary Biology, Harvard University, Cambridge, MA USA; 58 Museum of Comparative Zoology, Harvard University, Cambridge, MA USA; 59 Informatics Group, Harvard University, Cambridge, MA USA; 60 Sylvan Heights Bird Park, Scotland Neck, NC USA; 61 GenPhySE, INRA, INPT, INP-ENVT, Université de Toulouse, Castanet-Tolosan, France; 62 Laboratório Nacional de Computação Científica, Petrópolis, Brazil; 63 Department of Ecology and Evolutionary Biology, University of California Santa Cruz, Santa Cruz, CA USA; 64 Howard Hughes Medical Institute, University of California Santa Cruz, Santa Cruz, CA USA; 65 Institut de Recerca de la Biodiversitat (IRBio), Universitat de Barcelona, Barcelona, Spain; 66 Departament de Biologia Evolutiva, Ecologia i Ciències Ambientals (BEECA), Universitat de Barcelona, Barcelona, Spain; 67 Departament de Genètica, Microbiologia i Estadística, Universitat de Barcelona, Barcelona, Spain; 68 Department of Molecular, Cellular and Biomedical Sciences, University of New Hampshire, Durham, NH USA; 69 Department of Biology, Queen’s University, Kingston, Ontario Canada; 70 Department of Bioinformatics and Genetics, Swedish Museum of Natural History, Stockholm, Sweden; 71 Centre for Palaeogenetics, Stockholm, Sweden; 72 Milner Centre for Evolution, University of Bath, Bath, UK; 73 Instituto de Ecologia, UNAM, Mexico City, Mexico; 74 State Key Laboratory of Biocontrol, School of Ecology, Sun Yat-sen University, Guangzhou, China; 75 Center for Conservation Genomics, Smithsonian Conservation Biology Institute, Smithsonian Institution, Washington, DC USA; 76 New York Genome Center, New York, NY USA; 77 Department of Anatomy, University of Otago, Dunedin, New Zealand; 78 AG Neurosensory Sciences, Institut für Biologie und Umweltwissenschaften, University of Oldenburg, Oldenburg, Germany; 79 Biology Department, Texas A&M University, College Station, TX USA; 80 MPRG Behavioural Genomics, Max Planck Institute for Evolutionary Biology, Plön, Germany; 81 Institute of Clinical Molecular Biology, Christian-Albrechts-University of Kiel, Kiel, Germany; 82 Department of Wildlife Diseases, Leibniz Institute for Zoo and Wildlife Research, Berlin, Germany; 83 Environmental Resilience Institute, Indiana University, Bloomington, IN USA; 84 National Museum of Natural Sciences, Spanish National Research Council (CSIC), Madrid, Spain; 85 Department of Biology, Indiana University, Bloomington, IN USA; 86 Institute of Marine Science, University of Auckland, Auckland, New Zealand; 87 Center for Genomics and Systems Biology, Department of Biology, New York University – Abu Dhabi, Abu Dhabi, UAE; 88 Wildlife and Ecology Group, Massey University, Palmerston North, New Zealand; 89 School of Fundamental Sciences, Massey University, Palmerston North, New Zealand; 90 Departamento de Bioquímica e Biologia Molecular, Centro de Ciências, Universidade Federal do Ceará, Fortaleza, Brazil; 91 Laboratório de Genômica e Biodiversidade, Instituto de Bioquímica Médica Leopoldo de Meis, Rio de Janeiro, Brazil; 92 Department of Biology, University of Nevada Reno, Reno, NV USA; 93 Department of Integrative Biology and Physiology, UCLA, Los Angeles, CA USA; 94 Smithsonian Tropical Research Institute, Panama City, Panama; 95 Department of Wildlife Ecology and Conservation, University of Florida, Gainesville, FL USA; 96 Center for Latin American Studies, University of Florida, Gainesville, FL USA; 97 Department of Biology, George Mason University, Fairfax, VA USA; 98 Department of Biology and Neuroscience Minor, University of Mississippi, University, MS USA; 99 Department of Ecology and Evolutionary Biology, Brown University, Providence, RI USA; 100 Max Planck Institute for Ornithology, Seewiesen, Germany; 101 Department of Vertebrate Zoology, National Museum of Natural History, Smithsonian Institution, Washington, DC USA; 102 Behavior, Ecology, Evolution and Systematics Program, University of Maryland, College Park, MD USA; 103 Computational Biology Department, Carnegie Mellon University, Pittsburgh, PA USA; 104 Migratory Bird Center, Smithsonian National Zoological Park and Conservation Biology Institute, Washington, DC USA; 105 Department of Evolutionary Biology and Environmental Studies, University of Zurich, Zurich, Switzerland; 106 Biology Department, Boston College, Chestnut Hill, MA USA; 107 Department of Evolution, Ecology, and Behavior, School of Integrative Biology, University of Illinois at Urbana-Champaign, Urbana, IL USA; 108 International Research Center for Neurointelligence, University of Tokyo, Tokyo, Japan; 109 Museum of Southwestern Biology, Department of Biology, University of New Mexico, Albuquerque, NM USA; 110 Museum of Vertebrate Zoology, Department of Integrative Biology, University of California, Berkeley, Berkeley, CA USA; 111 National Center for Genome Resources, Santa Fe, NM USA; 112 Department of Zoology and Physiology, University of Wyoming, Laramie, WY USA; 113 School of BioSciences, The University of Melbourne, Melbourne, Victoria Australia; 114 Department of Ecology and Evolutionary Biology, University of Colorado Boulder, Boulder, CO USA; 115 Finnish Museum of Natural History, University of Helsinki, Helsinki, Finland; 116 Department of Behavioral Neuroscience, Oregon Health and Science University, Portland, OR USA; 117 Department of Biological Sciences, University of Toronto Scarborough, Toronto, Ontario Canada; 118 James D. Watson Institute of Genome Sciences, Hangzhou, China; 119 Center for Excellence in Animal Evolution and Genetics, Chinese Academy of Sciences, Kunming, China; 120 Danish Institute for Advanced Study, University of Southern Denmark, Odense, Denmark; 121 Institute of Ecology, Peking University, Beijing, China; 122 Department of Life Sciences, Imperial College London, Ascot, UK; 123 University Museum, Norwegian University of Science and Technology, Trondheim, Norway; 124 The Rockefeller University, New York, NY USA; 125 Howard Hughes Medical Institute, Chevy Chase, MD USA

**Keywords:** Evolutionary genetics, Comparative genomics

## Abstract

Whole-genome sequencing projects are increasingly populating the tree of life and characterizing biodiversity^1–4^. Sparse taxon sampling has previously been proposed to confound phylogenetic inference^5^, and captures only a fraction of the genomic diversity. Here we report a substantial step towards the dense representation of avian phylogenetic and molecular diversity, by analysing 363 genomes from 92.4% of bird families—including 267 newly sequenced genomes produced for phase II of the Bird 10,000 Genomes (B10K) Project. We use this comparative genome dataset in combination with a pipeline that leverages a reference-free whole-genome alignment to identify orthologous regions in greater numbers than has previously been possible and to recognize genomic novelties in particular bird lineages. The densely sampled alignment provides a single-base-pair map of selection, has more than doubled the fraction of bases that are confidently predicted to be under conservation and reveals extensive patterns of weak selection in predominantly non-coding DNA. Our results demonstrate that increasing the diversity of genomes used in comparative studies can reveal more shared and lineage-specific variation, and improve the investigation of genomic characteristics. We anticipate that this genomic resource will offer new perspectives on evolutionary processes in cross-species comparative analyses and assist in efforts to conserve species.

## Abstract (web summary)

A dataset of the genomes of 363 species from the Bird 10,000 Genomes Project shows increased power to detect shared and lineage-specific variation, demonstrating the importance of phylogenetically diverse taxon sampling in whole-genome sequencing.

## Main

Comparative genomics is rapidly growing, fuelled by the advancement of sequencing technologies. Many large-scale initiatives have been proposed with a core mission of producing genomes for hundreds of species, representing the phylogenetic diversity of particular taxa^6–8^. Although the generation of genomes is now more routine, an immediate challenge is how to efficiently compare large numbers of genomes in an evolutionary context. A critical first step is the accurate detection of orthologous sequences. In this study, we release a large-scale dataset of bird genomes, which we use to establish a framework for comparative analysis. We provide insight on how scaling-up genome sampling assists in our understanding of avian genomic diversity and in the detection of signals of natural selection down to individual bases.

**Fig. 1:**
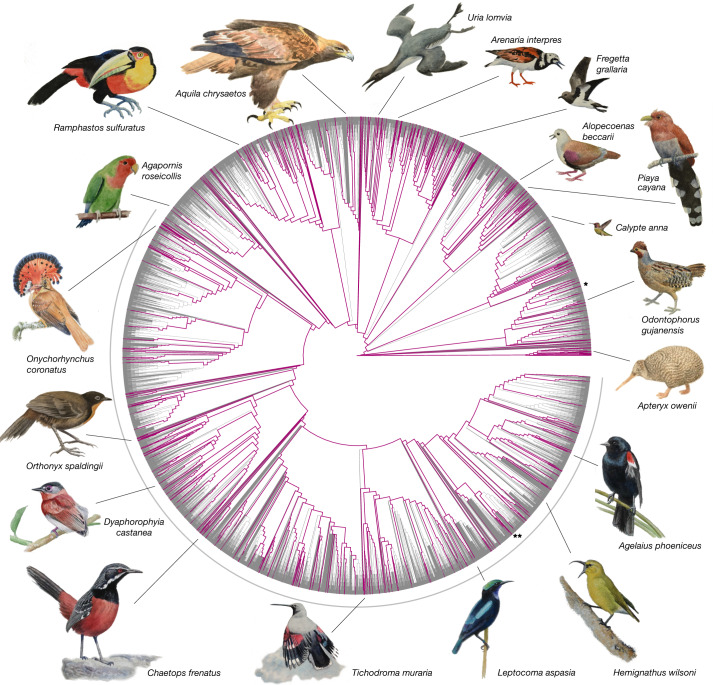
Newly sequenced genomes densely cover the bird tree of life. The 10,135 bird species^11,12^ are shown on a draft phylogeny that synthesizes taxonomic and phylogenetic information^36^ (Supplementary Data). In total, 363 species, covering 92.4% of all families, now have at least 1 genome assembly per sequenced family (purple branches). The grey arc marks the diverse Passeriformes radiation, with 6,063 species, of which 173 species have genome assemblies now. Chicken (*) and zebra finch (**) are marked for orientation. Paintings illustrate examples of sequenced species.

**Extended Data Fig. 1:**
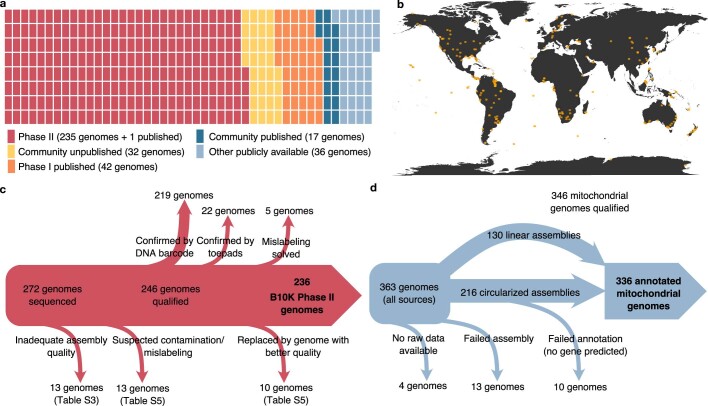
Sampling and processing of the 363 genomes. **a**, Sources of the 363 genomes. Each genome is a square; colour indicates the data source. Newly published genomes from the B10K Project phase II are red; unpublished genomes contributed by external labs are yellow; published genomes from phase I are orange; genomes contributed by the community that have since been published are dark blue; and other genomes available on NCBI are light blue. **b**, Map^63^ of geographical origin of the 281 bird samples for which geographical coordinates are available. **c**, Summary of the species confirmation of 236 B10K Project newly sequenced species. The downward arrows are excluded genomes. **d**, Summary of mitochondrial genome assembly and annotation for 336 species. The downward arrows are excluded mitochondrial genomes.

The B10K Project began with the Avian Phylogenomics Consortium (phase I), which analysed 48 genomes from representatives of most bird orders^9,10^. Here we report the genome sequencing outcomes from phase II of the project: these outcomes include a total of 363 species in 92.4% (218 out of 236^11,12^) of avian families (Supplementary Tables 1–5). Species were selected to span the overall diversity and to subdivide long branches, when possible (Fig. 1, Supplementary Data). Our sampling covers bird species from every continent (Extended Data Fig. 1) and more than triples the previous taxonomic coverage of avian genome sequencing; to our knowledge, 155 bird families are represented here for the first time. We chose short-read sequencing as our main strategy for generating data, which enabled us to use older samples (the oldest of which was collected in 1982) and access rare museum specimens—such as one of the few vouchered tissues of the Henderson crake (*Zapornia atra*), which occurs on a single island. We incorporated 68 species of concern on the International Union for Conservation of Nature (IUCN) Red List of Threatened Species (Supplementary Table 1); these include 12 endangered and 2 critically endangered species—the plains-wanderer (*Pedionomus torquatus*) and the Bali myna (*Leucopsar rothschildi*, which has fewer than 50 adults remaining in the wild^13^).

Two hundred and sixty-seven of the 363 species represented in our genome data are newly released, comprising 18.4 trillion base pairs (bp) of raw data and 284 billion bp of assemblies. The assemblies are comparable in quality to previously published bird genomes^9,10^, but vary in contiguity (average scaffold N50 = 1.42 megabases (Mb), contig N50 = 42.57 kilobases (kb); see interactive supplementary figure 1, hosted at https://genome-b10k.herokuapp.com/main). The sequencing coverage ranged from 35× (blue-throated roller (*Eurystomus gularis*) and yellowhead (*Mohoua ochrocephala*)) to 368× (song sparrow (*Melospiza melodia*)) and genomic completeness was high (average 95.8%). We annotated all 363 genomes using a homology-based method with a uniform gene set that included gene models from chicken, zebra finch and human, and published transcriptomes (Supplementary Tables 6–8), to predict an average of 15,464 protein-coding genes for each species (Supplementary Table 1). We also assembled mitochondrial genomes for 336 species, with 216 species fully circularized and 228 species with a complete mitochondrial annotation (Supplementary Table 1).

**Extended Data Fig. 2:**
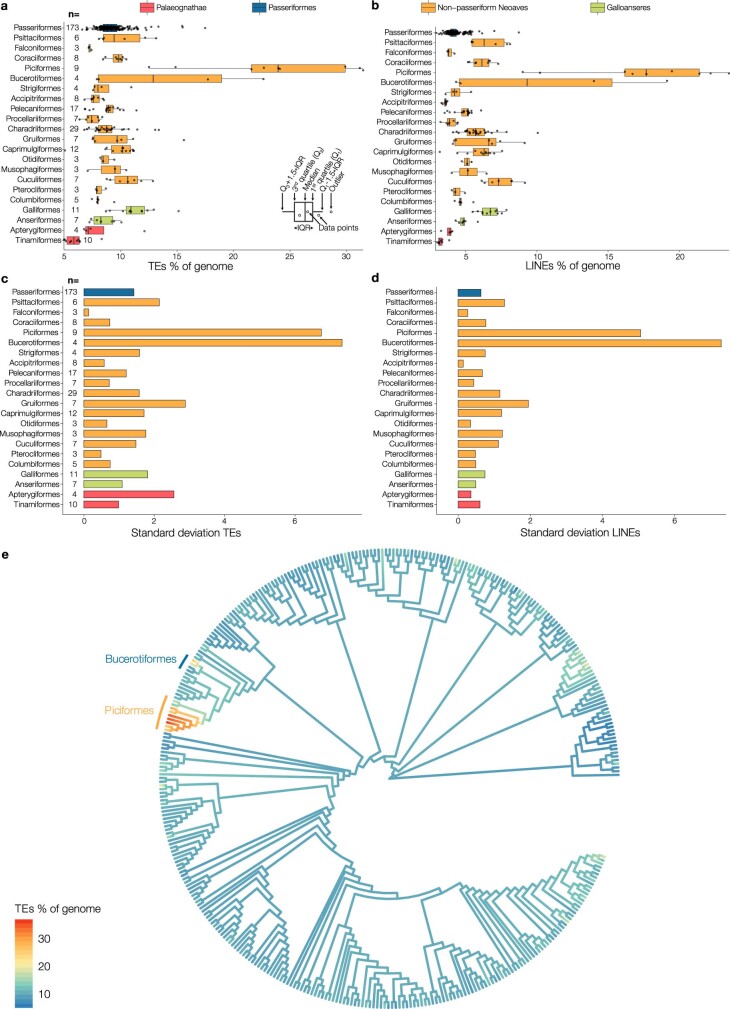
Distribution of transposable elements. **a**, Percentage of the genome that is a transposable element (TE). Box plots are shown for groups with at least three sequenced species. **b**, Per cent base pairs of the genome that are long interspersed nuclear elements (LINEs), grouped by orders. Box plots are shown for groups with at least three sequenced species. **c**, S.d. of the transposable element content for orders with at least three sequenced species. **d**, S.d. of the per cent LINE content for orders with at least three sequenced species. **e**, Ancestral state reconstruction of total transposable elements. The branch colour from blue to red indicates an increase in transposable elements. Two orders with noticeable patterns—Piciformes and Bucerotiformes—are labelled on the tree. A zoomable figure with labels for all terminals is available at www.doi.org/10.17632/fnpwzj37gw.

Bird genomes at the ordinal level were previously found to contain a low proportion of transposable elements, except for the downy woodpecker (*Picoides pubescens*) in Piciformes^10^. Consistent with these findings, 96.1% of birds at the family level had a transposable element content lower than 15%—but we found additional outliers (Extended Data Fig. 2a, Supplementary Table 1). In particular, long interspersed nuclear elements were prevalent in all nine sequenced species in Piciformes, which suggests an ancestral expansion in this lineage (24% on average, Welch two-sample *t*-test, *P* = 9.98 × 10^−5^) (Extended Data Fig. 2b, d). The common scimitarbill (*Rhinopomastus cyanomelas*) and common hoopoe (*Upupa epops*) in Bucerotiformes also had exceptionally high transposable element content (23% and 18%, respectively) owing to recent expansions of long interspersed nuclear elements, whereas two hornbill species in the same order exhibited the typical low proportions (Extended Data Fig. 2e).

Previous studies have suggested that hundreds of genes were lost in the ancestor of birds^10,14^. Gene-loss inference is complicated by incomplete assemblies and can be unreliable with only a few species^15^. We found that 142 genes previously considered to be absent in Aves^10^ were detected in at least one of the newly sequenced bird genomes (Supplementary Table 9), which implies that these genes were either lost multiple times or missed in the assemblies of the 48 birds of B10K Project phase I. Nonetheless, 498 genes remained absent across all 363 bird species, which adds to evidence that these genes were truly lost in the common ancestor.

**Extended Data Fig. 3:**
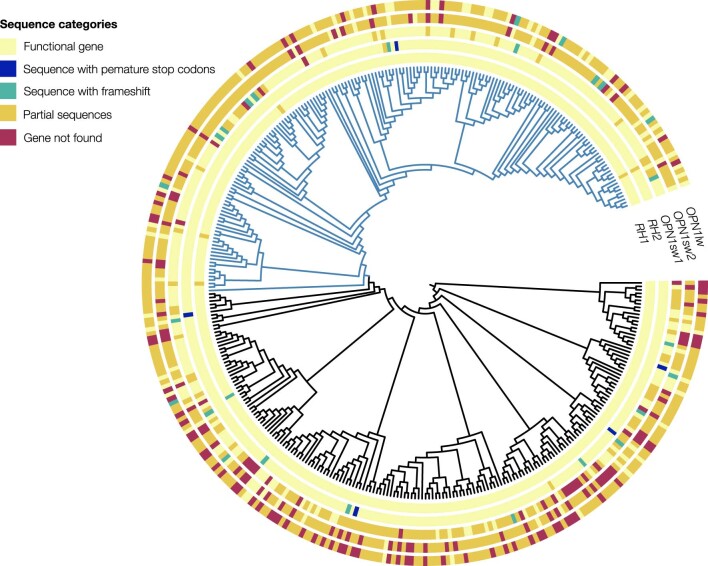
Patterns of the presence and absence of 5 visual opsins in 363 bird species. This figure shows patterns for the visual opsins encoded by *RH1*, *RH2*, *OPN1sw1*, *OPN1sw2* and *OPN1lw*. Colours correspond to five annotated states of opsin sequences. A zoomable figure with labels for all terminals is available at www.doi.org/10.17632/fnpwzj37gw.

We also investigated a number of genes that were previously associated with phenotypes and physiological pathways. For example, we found that rhodopsin (encoded by *RH1*) and the medium-wavelength sensitive opsin (encoded by *RH2*) were present in all 363 birds, but were incomplete or pseudogenized in 5 and 11 species, respectively (Supplementary Table 10). The other three conopsin genes showed a more varied pattern of presence and absence. *OPN1sw2* and *OPN1lw* existed either as partial sequences or were completely absent in 310 and 308 species, respectively, and *OPN1sw1* was functional in more than half of the 363 birds—especially in Passeriformes (perching birds) (Extended Data Fig. 3, Supplementary Table 10).

**Extended Data Fig. 4:**
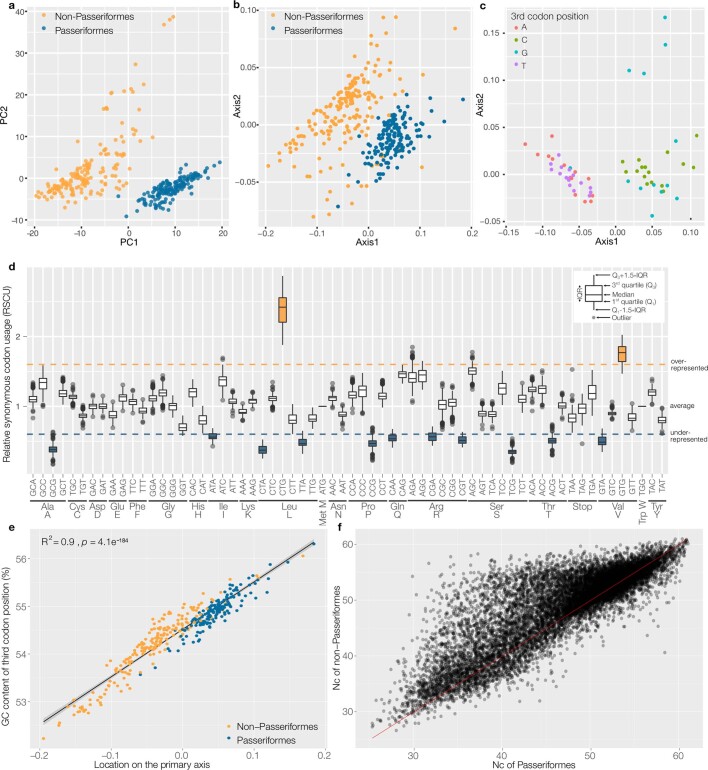
GC content and codon use. **a**, Principal component analysis (PCA) of GC content in the coding regions of orthologues with conserved synteny with chicken for 340 bird species, including 164 Passeriformes species. **b**, Correspondence analysis of RSCU for all 363 birds. The primary and secondary axes account for 78.18% and 14.82% of the total variation, respectively. **c**, The distribution of codons on the same two axes as shown in **b**, with each codon coloured according to its ending nucleotide. This showed that the axis-1 score of a species is primarily determined by differences in frequencies of codons ending in G, C, A or T. **d**, RSCU analysis of 59 codons across avian genomes (*n* = 363 biologically independent species for each box plot). The horizontal lines indicate thresholds of under-represented codons (<0.6, blue box plots), average representation (1.0, white box plots) and over-represented codons (>1.6, orange box plots). **e**, Pearson correlation between GC content of the third codon position and the primary axis in **b**, colour-coded to distinguish Passeriformes and non-Passeriformes. The strong correlation (*R*^2^ = 0.9, *P* = 4.1 × 10^−184^) indicates that the frequencies of codons ending in G or C is the main driver of the codon bias in Passeriformes. **f**, Comparison of the mean Nc values between the Passeriformes and other species for orthologues with conserved synteny with chicken (Supplementary Table 12). Each dot represents the mean Nc value of an orthologue in the Passeriformes and other species, respectively. Orthologues with at least 20 individuals in both the Passeriformes and the non-Passeriformes were included in this analysis.

Passeriformes also had a notably higher GC content than other birds in coding regions (Welch two-sample *t*-test, *P* = 7.59 × 10^−43^) (Extended Data Fig. 4a) but not in non-coding regions (Welch two-sample *t*-test, *P* = 0.06). Differences in GC content can result in biased use of particular synonymous codons over others, which can affect gene expression and translation efficiency^16^. Consistent with this hypothesis, relative synonymous codon use values for 59 synonymous codons (excluding non-degenerate codons, Met, Trp and three stop codons) showed substantial differences between Passeriformes and other birds, especially in the preference of codons ending in G or C (Extended Data Fig. 4b, c, e). Passeriformes significantly deviated from random use of synonymous codons with a smaller average effective number of codons compared to other birds (paired-sample *t*-test, *P* < 2.2 × 10^−16^) (Extended Data Fig. 4f). These results indicate that the GC content may have affected the gene evolution of the speciose Passeriformes.

To gain further evolutionary insight from the genomes, we constructed a whole-genome alignment of the 363 genomes using a progressive version of the reference-free aligner Cactus^17,18^. Cactus produced a substantially more-complete alignment than the commonly used reference-based method MULTIZ^19^, particularly when the aligned species were phylogenetically distant from the chicken reference^17^. In comparison to a previous alignment of the 48 bird genomes using chicken and zebra finch as references^10^, our reference-free approach and extended sampling unlocked a far greater proportion of orthologous sequences: 981 Mb across the whole genome (a 149% increase), 24 Mb of orthologous coding sequence (an 84.4% increase) and 141 Mb of orthologous introns (a 631% increase) that derived from the common avian ancestors between chicken and any other bird species.

**Fig. 2:**
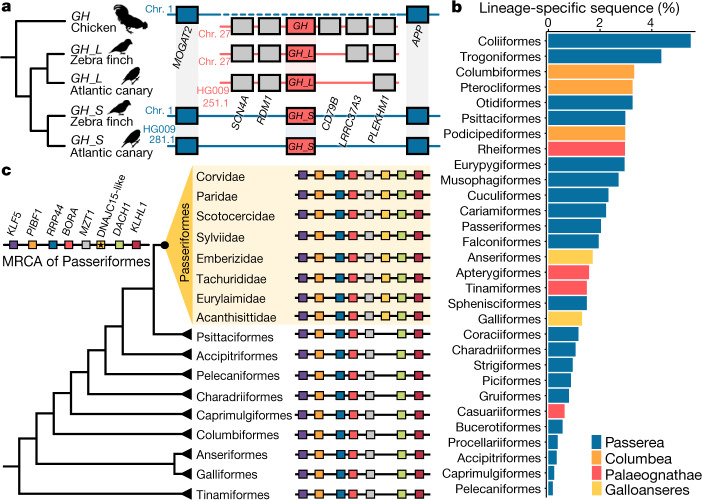
Improved orthologue distinction and detection of lineage-specific sequences. **a**, Incorporating synteny in the orthologue assignment pipeline resolves complex cases of orthology. The growth hormone gene (*GH*) has one copy in chicken and two copies in Passeriformes (exemplified by zebra finch and Atlantic canary). On the basis of the conserved synteny of the *GH_L* in Passeriformes with *GH* of chicken, the pipeline recognized *GH_L* as the ancestral copy—despite high similarity to the other copy. **b**, The whole-genome alignment allows detecting lineage-specific sequences. For orders with more than one sequenced representative, lineage-specific sequences are those present in the reconstructed ancestral genome but absent in other lineages. Colours denote higher-level taxonomic groupings^9^. **c**, A novel gene in Passeriformes. Phylogeny based on the B10K Project phase I^9^ plotted with synteny of a putative lineage-specific gene (*DNAJC15L*) and its surrounding genes. *DNAJC15L* is found in 131 out of 173 sequenced Passeriformes and their reconstructed ancestral genome, but is not found in non-Passeriformes. MRCA, most-recent common ancestor.

**Extended Data Fig. 5:**
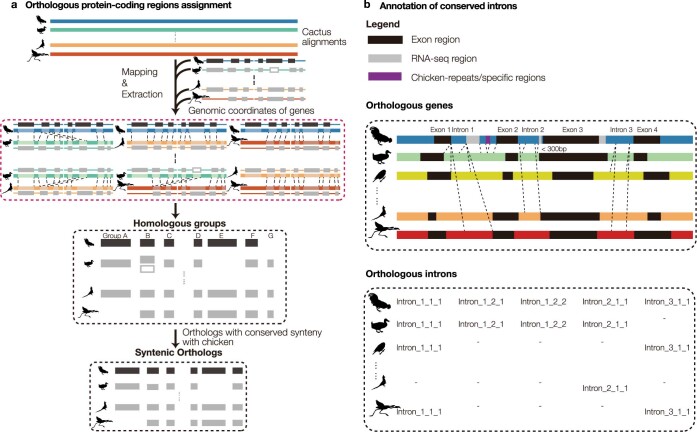
Overview of the pipelines for identifying genomic regions. **a**, Assignment of orthologous protein-coding regions. All pairwise relationships between homologous regions obtained from the Cactus alignment (4 species shown here in different colours) were used to construct the homologous groups across all 363 birds. Using chicken as the reference, we further generated a table containing homologues with conserved synteny to chicken. **b**, Annotation of conserved orthologous intron regions on the basis of Cactus whole-genome alignments. The credible intron fragments in chicken were picked out after filtering out regions mapped by RNA sequences, and chicken-specific or repetitive regions. Orthologous relationships of intron fragments were detected on the basis of the aligned Cactus hits and the orthologues with conserved synteny with chicken. The non-intron regions of each bird in the alignments were masked as gaps.

**Extended Data Fig. 6:**
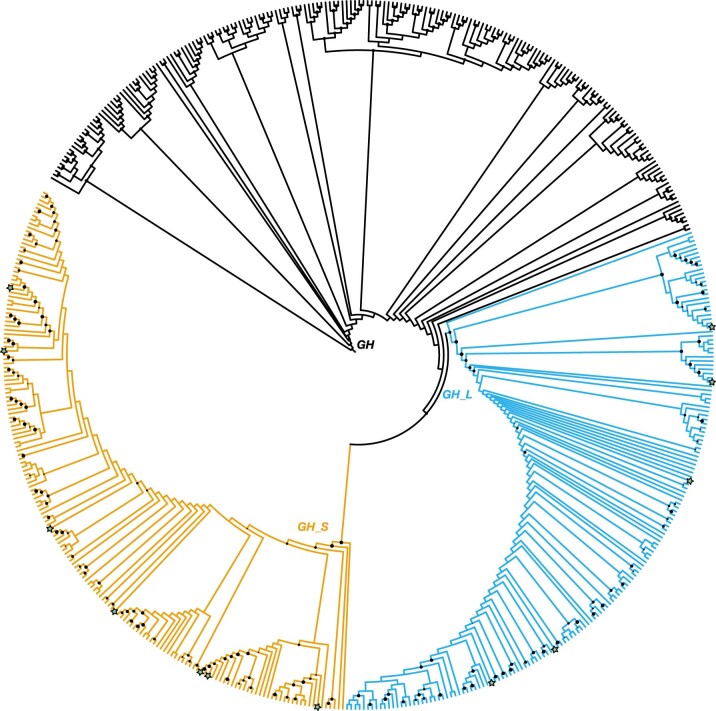
Gene tree for copies of the growth hormone gene *GH*. The tree was generated by maximum likelihood phylogenetic analysis^64^ of avian *GH* gene copies. Only nodes with >80 bootstrap are annotated as dots; the larger the dot, the higher the bootstrap. All Passeriformes sequences are clustered in a single clade and there are two sister gene clades within Passeriformes, corresponding to the *GH_S* gene copy (blue) and the *GH_L* gene copy (orange). Twelve species with only one copy are indicated by green stars. A zoomable figure with labels for all terminals and the tree file is available at www.doi.org/10.17632/fnpwzj37gw.

Gene duplications are an important mechanism that shapes genome evolution, because duplicated copies often evolve under different selective pressures and evolutionary rates^20^. We developed an orthologue assignment pipeline that incorporates information about the genomic context of the gene copies (synteny) with the Cactus alignment to permit distinguishing between the ancestral copies, those inherited from a more recent common ancestor and duplicated novel copies (Extended Data Fig. 5a, Supplementary Tables 11, 12). An example is the growth hormone (*GH*) gene that was previously found to be duplicated in 24 Passeriformes (to produce *GH_L* and *GH_S*)^21^. We confirmed that this gene duplication occurred exclusively in Passeriformes (found in 161 out of 173 species; its absence in 12 species is caused by incomplete assemblies), resulting in a one-to-many relationship with the single copy in other birds (Extended Data Fig. 6). The synteny with surrounding genes identified the passeriform *GH_L* as the ancestral copy, and *GH_S* as a newly derived copy located in a different genomic context (Fig. 2a). Moreover, when the pipeline was applied to both datasets (of 48 and 363 bird species), the higher taxon sampling allowed the detection of 439 additional orthologues with conserved synteny to chicken—many of which were lineage-specific gene copies. These additional orthologues, improved by the denser representation of species and the Cactus alignment, will drive downstream comparative analyses.

**Extended Data Fig. 7:**
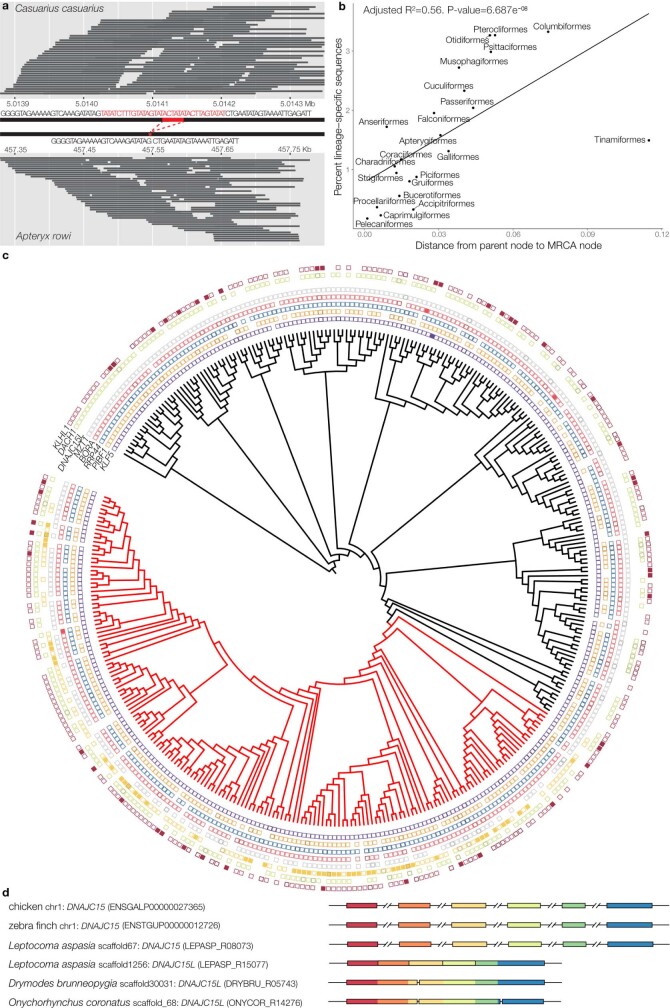
Identification of lineage-specific sequences. **a**, An example of a 36-bp insertion (red) identified by Cactus in the southern cassowary (*Casuarius casuarius*) compared to the Okarito brown kiwi (*Apteryx rowi*) (both in Palaeognathae) with mapped sequence reads shown as lines. **b**, Proportion of lineage-specific sequence for each order correlated with the distance from parent node to MRCA node (branch length). **c**, Presence and absence of the DNAJC15-like gene (*DNAJC15L*), and its surrounding genes, in all 363 birds. Upstream: *KLHL1* and *DACH1*; downstream: *MZT1*, *BORA*, *RRP44*, *PIBF1* and *KLF5*. The state is shown for each bird in three ways: multiple copies (filled shapes), one copy (empty shapes) and no gene (blank). Passeriformes are highlighted in red. A zoomable figure with labels for all terminals is available at www.doi.org/10.17632/fnpwzj37gw. **d**, Exon fusion patterns of the DNAJC15-like gene (*DNAJC15L*) in three Passeriformes, compared to exon structure of the ancestral *DNAJC15*. For *L. aspasia*, gene models for the ancestral and novel copy are shown. The structure of the ancestral copy is highly conserved across all bird species with five introns. The Passeriformes-specific copy has no intron or newly derived minor intron and includes a poly-(A) at the 5′ end, which implies that this new gene was derived from retroduplication of *DNAJC15*.

Using the Cactus alignment, we reconstructed an ancestral genome for each evolutionary node to characterize both shared and lineage-specific genomic diversity. Being able to identify sequences unique to particular lineages, and not only those shared with a reference genome, is a major advantage of a reference-free alignment^22^. We found that lineage-specific sequences constitute 0.2% to 5.5% of the reconstructed genome of the most-recent common ancestor of each order (Fig. 2b, Supplementary Table 13). Among these, we identified 154 Passeriformes-specific genes (Supplementary Table 14). The gene present in the largest number of passerines (131 out of 173 species) is a paralogue of the heat shock protein gene *DNAJC15*, which has many copies in bird genomes and is thought to be associated with the biogenesis of mitochondria^23^ and fertilization^24^. We identified a novel Passeriformes-specific copy (which we named DNAJC15-like (*DNAJC15L*)) at a newly derived genomic region between the *MZT1* and *DACH1* genes (based on the chicken coordinates), which was reconstructed as a duplication in the most-recent common ancestor of Passeriformes (Fig. 2c, Extended Data Fig. 7c). The *DNAJC15L* gene model showed exon fusions compared to its parental gene, which suggests that a retrotransposition mechanism was the probable origin of this duplication (Extended Data Fig. 7d).

**Extended Data Fig. 8:**
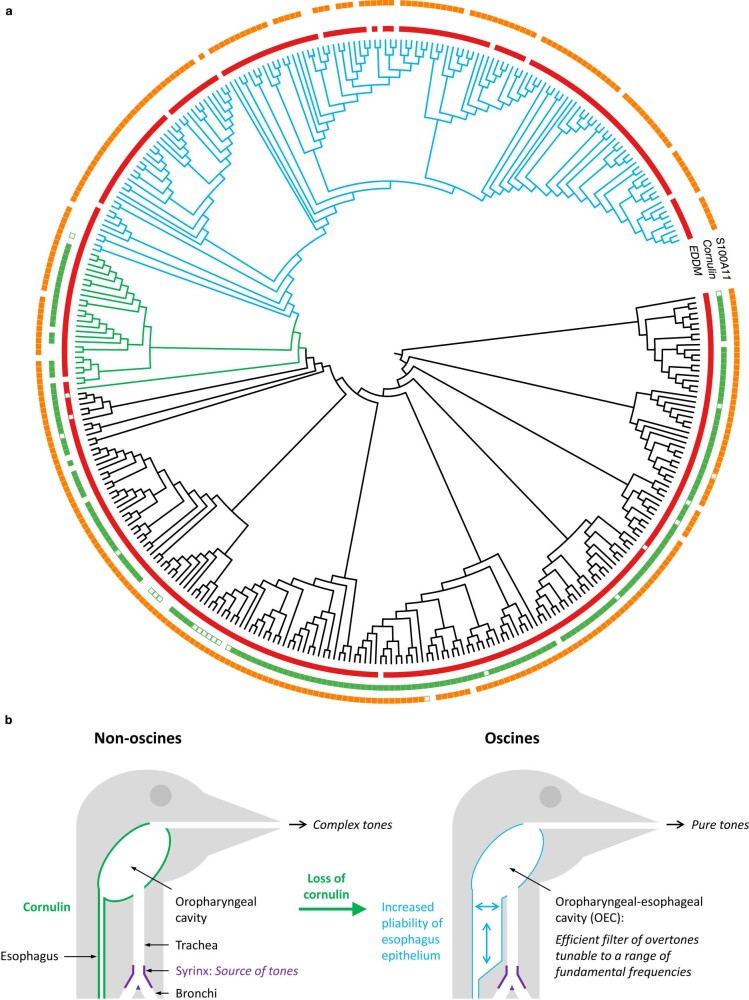
The evolution of songbirds was associated with the loss of the cornulin gene. **a**, Presence and absence of the cornulin gene (*CRNN*) and its surrounding genes (*EDDM* and *S100A11*) in all 363 birds. Branches are coloured as oscine Passeriformes (blue), non-oscine Passeriformes (green) and non-Passeriformes (black). The states of genes are shown in three ways: functional gene (filled box), pseudogene (empty box) and gene not found (blank). Genes were identified by Exonerate^65^ using phylogenetically diverse *EDDM*, *CRNN* and *S100A11* sequences as queries. A zoomable figure with labels for all terminals is available at www.doi.org/10.17632/fnpwzj37gw. **b**, Hypothesis on the evolutionary loss of cornulin and the appearance of a fine-tuned extensibility of the oesophagus as a vocal tract filter in songbirds.

Moreover, we identified lineage-specific losses of genes such as cornulin (*CRNN*), which encodes a prominent structural protein of the oesophageal and oral epithelium in humans and chicken^25^. This gene is disrupted by mutations or is entirely absent in Accipitriformes (eagles and related birds of prey), Phalacrocoracidae (cormorants) and Passeri (songbirds, a group of Passeriformes) (Extended Data Fig. 8a). The latter use rapid changes in the diameter of the upper oesophagus to tune their vocal tract to the fundamental frequency of their song^26^. The absence of *CRNN* might correspond to changes in visco-elastic properties of the oesophageal epithelium, and the loss of this gene may have contributed to the evolution of the diverse pure-tone vocalizations of songbirds (Extended Data Fig. 8b).

We next explored avian conserved sequences, genomic regions that evolve at a substantially slower substitution rate than expected under neutral evolution. Conserved sequences are often indicators of purifying selection^27^ and are therefore useful for investigating function within the genome^28^. To identify and measure conserved regions, we created conservation scores for each base pair of the 363-species Cactus alignment projected onto the chicken genome. The dense sampling increased our ability to detect purifying selection enormously, and allowed us to produce what is—to our knowledge—the first base-by-base conservation annotation that covers a substantial portion of a bird genome. We scaled our model of the genome-wide mutation rate to match the neutral rate observed in microchromosomes, macrochromosomes and sex chromosomes, because each chromosome type shows different evolutionary rates in birds^29,30^. This resulted in one model for each chromosome type, which together were then used to evaluate the degree of departure from the neutral rate and to estimate the conservation score for each site. With the 363-way data, we found that the neutral rate within sex chromosomes is 16% faster than in macrochromosomes, and that the neutral rate within macrochromosomes is 9% faster than in microchromosomes.

**Fig. 3:**
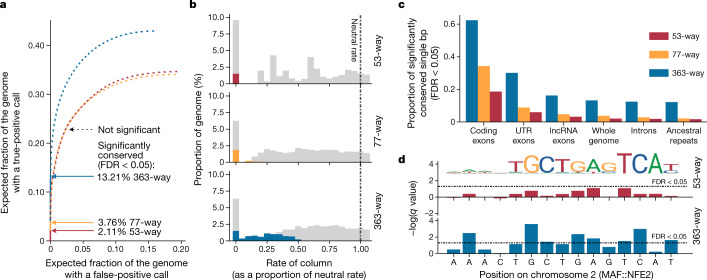
Denser phylogenomic sequencing increases the power to detect selective constraints. Results are shown from 3 alignments for 53 birds, 77 vertebrates, and 363 birds. **a**, Proportion of alignment columns labelled as conserved. The cumulative portion of the genome with a conserved call is shown, starting from the column with the smallest *P* value and proceeding to the columns with the highest *P* values. The dotted lines show the path after hitting the false-discovery rate (FDR) *P* value cutoff of 0.05, below which calls are significant (marked by arrows). **b**, Histograms of the rate of alignment columns evolving slower relative to the neutral rate (labelled 1.00). Coloured areas indicate significantly conserved columns, and light grey areas indicate non-significantly conserved columns. A rate of zero contains a relatively high proportion of recent insertions present in only a few species; there is limited statistical power to classify such insertions. **c**, Proportion of various functional regions of the chicken genome that contain single-bp conserved elements in the large alignment compared to alignments with fewer species. UTR, untranslated region. **d**, An example of a MAF::NFE2 motif overlaid on one of its predicted binding sites demonstrates the high resolution of our conserved site predictions and the increased power to predict conservation in the larger alignment.

We compared these results against conservation scores derived from two smaller alignments: a MULTIZ 77-way alignment including birds and other vertebrate outgroups^31^, and a 53-way alignment containing only birds of the 77-way alignment. A previous comparison of 48 bird genomes found that at least 7.5% of the chicken genome was conserved, with significantly lower substitution rates than the background^10^. This ratio was reached at 10-bp resolution by integrating across multiple adjacent bases, trading off a lower resolution for a necessary increase in statistical power. This is because the statistical power to detect conserved elements is roughly proportional to the total branch length between the aligned species^32^. Our reference-free alignment of 363 bird species resulted in a predicted total branch length of 16.5 expected substitutions per site, compared to 9.9 within the 77-way and 4.3 within the 53-way alignments. We transformed the conservation scores into calls of significantly conserved single-base-pairs at an expected false-discovery rate^33^ of 5%. The 363-way alignment provided ample increases in the number of bases detectable as conserved relative to alignments that contain fewer taxa (13.2% of the chicken genome in the 363-way alignment versus 3.8% in the 77-way and 2.1% in the 53-way) (Fig. 3a, Supplementary Table 15). Such an improvement cannot be explained by the alignment method, as a Cactus 48-way alignment of birds showed very similar results to the 53-way MULTIZ alignment (Extended Data Fig. 10d). In the Z chromosome (which has a generally faster evolutionary rate than other chromosomes), we detected 8.4 Mb (10.2%) of the chromosome as significantly conserved in the 363-way alignment—8.8-fold higher than in the 53-way alignment.

These results offer increased power to detect weakly conserved regions (that is, regions that exhibit mutations but at lower than the neutral rate). Detectable weakly conserved regions evolved at a maximum of 52% of the neutral rate according to the 363-way alignment, compared to only 26% for the smaller 77-way alignment (Fig. 3b). The 53-way alignment provided power only to detect conserved bases that were completely unchanged across all sampled birds. The 363-way alignment detected 62.4% of bases within coding exons as conserved (74.7% for the first 2 codon positions), higher than the 34.3% within the 77-way alignment and the 18.6% within the 53-way alignment (Fig. 3c). Furthermore, the increase was proportionally much larger in functional non-coding regions of the genome, including bases within long non-coding RNAs (lncRNAs) (16.2% versus 4.8% and 3.2%), untranslated exons (30.1% versus 8.8% and 6.0%) (Fig. 3c), and other regulatory regions such as transcription factor binding sites (51.2% versus 9.7% and 6.9%) (Fig. 3d). Taken together, our results suggest that although functional non-coding regions are more plastic and less strongly conserved than coding regions, much of their sequence is under a higher degree of selective constraint than previously realized with sampling using fewer taxa.

Overall, our dataset establishes birds as a system with unparalleled genomic resources. The B10K consortium is using these genomes and alignments to reconstruct the evolutionary history of birds, and the genomic patterns that underlie the diversity of avian phenotypes^34,35^. The genomes will further serve the community in two ways. Individually, the genomes can be used to investigate species-specific traits and to support conservation efforts of the sequenced species and their relatives. Collectively, the genomes and their alignments facilitate cross-species comparisons to gain new perspectives on evolutionary processes and genomic diversity.

## Methods

No statistical methods were used to predetermine sample size. The experiments were not randomized and investigators were not blinded to allocation during experiments and outcome assessment.

### Sample selection, DNA extraction, sequencing and assembly

A total of 363 species from 218 families were included. The 363 genomes came from 4 data sources, and included 267 newly sequenced genomes and 96 publicly available genomes (Extended Data Fig. 1a, Supplementary Table 1). A total of 236 genomes were sequenced specifically for this project, drawing on samples from 17 scientific collections. The 3 largest contributors were the National Museum of Natural History of the Smithsonian Institution (140 species), Louisiana State University Museum of Natural Science (31 species) and Southern Cross University (23 species). According to the tissue type, we used different commercial extraction kits following the manufacturers’ guidelines. B10K Project genomes were sequenced at BGI using the short-read sequencing strategy and assembled with SOAPdenovo v.2.04^37^ and Allpaths-LG v.52488^38^ (if applicable). Supplementary Table 1 provides summaries of assembly quality and BUSCO completeness assessment^39^. We conducted de novo assembly of the mitochondrial genomes using NOVOPlasty v.2.7.2^40^ and annotated them with MitoZ v.2.3^41^. Species identity was confirmed with mitochondrial and nuclear barcodes (Supplementary Tables 4–6). Detailed descriptions of these procedures are available in the Supplementary Information.

### Annotation of repeat and protein-coding genes

Tandem repeats were identified by Repeats Finder v.4.07b^42^ and transposable elements were annotated using both homology-based (RepeatMasker v.open-4.0.7^43^) and de novo (RepeatModeler v.open-1-0-8^44^) approaches. The ancestral state of total transposable elements was reconstructed with maximum likelihood using the fastAnc function in the R package phytools v.0.7-20^45^.

Annotation of protein-coding genes across the 363 bird genomes was conducted with a homology-based method using a primary reference gene set containing 20,194 avian genes (Supplementary Table 7). These annotations were then supplemented by non-redundant annotations from the 20,169 human gene set and the 5,257 transcriptomes set (Supplementary Information, Supplementary Table 8). Analyses of genetic and functional diversity of previously reported genes^10,21^, and of the cornulin gene in songbirds are described in the Supplementary Information.

### Cactus whole-genome alignment

We ran Cactus (at commit f88f23d) on the Amazon Web Services (AWS) cloud, using the AWSJobStore of Toil to store intermediate files. We generated a phylogenetic hypothesis to use as a guide tree for Cactus by extracting ultraconserved element regions^46^ from each of the 363 bird assemblies following a standard protocol^47^ (https://phyluce.readthedocs.io/en/latest/tutorial-three.html) and performed maximum likelihood inference on the concatenated dataset using ExaML v.3.0.9^48^ on an high-performance computing system, assuming a general time-reversible model of rate substitution, γ-distributed rates among sites and five tree searches (Supplementary Information). We used an auto-scaling cluster, which varied in size during the course of the alignment but used a combination of c3.8xlarge (high-CPU) and r3.8xlarge (high-memory) worker nodes. A MAF-format file was derived from this alignment using a parallelized version of the command hal2maf --onlyOrthologs --refGenome Gallus_gallus.

Chicken and zebra finch were marked as preferred outgroups (meaning that they would be chosen as outgroups if they were candidates), to ensure that a high-quality assembly was almost always available as an outgroup. Three genomes were used as outgroups to the avian tree: common alligator (*Alligator mississippiensis*) (v.ASM28112v4), green anole (*Anolis carolinensis*) (v.AnoCar2.0) and green sea turtle (*Chelonia mydas*) (v.CheMyd1.0). These outgroups were not included in the alignment, but used only to provide outgroup information for subproblems near the root (by using the --root option to select only the bird subtree).

### Orthologue identification

Definitions for the terms regarding homology and orthology that are used throughout the Article are based on previous publications^49,50^ and the resources of Ensembl (https://asia.ensembl.org/info/genome/compara/homology_types.html). Two genes are considered homologues if they share a common origin; that is, if they are derived from a common ancestor. A homologous group is a cluster of genes that evolved from one ancestor. Orthologues are homologous genes that result from a speciation event. Paralogues are homologous genes that result from a duplication event. A one-to-one orthologue is an orthologue of which only one copy is found in each species. A one-to-many orthologue occurs when one gene in one species is orthologous to multiple genes in another species. Many-to-many orthologues represent situations in which multiple orthologues are found in both species. In one-to-many and many-to-many orthologues, the gene copy that is located in a specific genomic context by synteny is identified as the ancestral copy. In one-to-many and many-to-many orthologues, the gene copy that is out of the genomic context (no synteny) is considered as the duplicated gene copy.

We identified orthologues using a synteny-based orthologue assignment approach that built on the whole-genome alignment generated by Cactus and synteny evidence (Extended Data Fig. 5a). All potential homologous groups were captured from the Cactus alignment without specifying a reference genome. To gain insight into the relationships among different copies of one-to-many and many-to-many orthologue groups, we further applied the synteny evidence to distinguish the ancestral and novel copy, using the following steps.

#### Data preparation

To obtain the putative homologous regions across all 363 species, we extracted the aligned protein-coding regions from HAL-format files of the Cactus pipeline, on the basis of the coordinate information of all the genes in each species.

#### Homologous group construction

The intersection of the putative homologous regions from the data preparation step and the coordinate information of the coding regions of protein-coding genes of each species from GeneWise predictions constituted the candidate homologous relationships between all possible pairs of species. All of these pairwise relationships were used to construct the homologous groups across all 363 bird species. To achieve this objective, we clustered all genes with the relevant pairwise relationship into a homologous group by setting the single-linkage clustering with minimum edge weight as zero (Supplementary Table 11).

#### Detection of ancestral and derived copies

The synteny evidence makes positional information valuable in distinguishing the ancestral and novel copy in one-to-many and many-to-many orthologues. For example, we could use the gene synteny between chicken and other species to identify the ancestral copy in the pairwise orthologues of chicken genes in any other species, which is the copy with the consistent synteny as in the chicken (Supplementary Table 12). This step refines the relationships using the synteny evidence to distinguish the ancestral and novel copies in one-to-many and many-to-many orthologues. The ancestral copy of a one-to-many orthologue is sometimes referred to as the strict orthologue or positional orthologue^51,52^.

#### Effect of adding species on orthologues with conserved synteny with chicken

To test whether adding species helps to identify more orthologues with conserved synteny with chicken, we also applied this method to the 48 birds analysed in phase I of the project.

### Intron dataset construction

Introns of the 15,671 orthologues among 363 species with conserved synteny with chicken were extracted from the Cactus alignment (Extended Data Fig. 5b). Detailed descriptions are available in the Supplementary Information.

### Codon preference

To examine the variation in codon usage, we conducted a correspondence analysis on the relative synonymous codon usage (RSCU) values^53^ at the species level and used the mean values of the effective number of codons (Nc)^54^ as an gene-level index to assess the differences between the Passeriformes and other species. Detailed descriptions are available in the Supplementary Information.

### Lineage-specific sequences on the basis of whole-genome alignments

We built a pipeline to identify lineage-specific sequences from Cactus alignments. We define lineage-specific sequences as sequences that occur only in the target lineage, do not align to the non-target lineages and that can be located in the reconstructed genome of the MRCA of the target lineage. Cactus reconstructs the ancestor sequences at each node according to the guide tree. By comparing the target lineage genome and its MRCA genome to their parent nodes on nodes deeper into the tree, we could identify newly emerged sequences of each MRCA and terminal branches as unaligned regions. Lineage-specific duplication with high similarity is not in the scope of this pipeline. Such lineage-specific duplication events need to be clarified by introducing synteny information, and our orthologue assignment pipeline has a good ability to distinguish these events (for example, the specific copy of *GH* in Passeriformes, as shown in Fig. 2a).

To obtain the total length of the lineage-specific sequences for all 37 avian orders, the reconstructed ‘genome’ of the MRCA of each order was mapped back to their parent nodes. Further, we investigated the correlation between the proportion of lineage-specific sequence and the distance from the MRCA node of each order to their immediate ancestor as a proxy of divergence time (with the branch length as estimated from 4D sites).

Passeriformes were used as an example to detect lineage-specific protein-coding genes. We identified all genes located in alignment regions that only appear in one of the Passeriformes lineages as putative Passeriformes-specific genes. To validate that these genes are truly Passeriformes-specific genes, we searched these genes using BLAST v.2.2.26 against all genes classified as non-Passeriformes genes and filtered any genes that had a reciprocal BLAST hit with non-Passeriformes. We also required that putative Passeriformes-specific genes evolved in the MRCA genome of Passeriformes, and therefore we mapped these genes to the reconstructed genome of the MRCA of Passeriformes. Any genes with less than 20 amino acid overlap in the protein-coding regions with the Passeriformes MRCA sequences were removed.

For the putative Passeriformes-specific gene that was present in the largest number of Passeriformes (DNAJC15-like, *DNAJC15L*), we investigated synteny with 7 flanking genes in all 363 birds (Extended Data Fig. 7c). We further examined patterns of exon fusion in the gene model for *DNAJC15L* in three Passeriformes (black sunbird (*Leptocoma aspasia*), southern shrub robin (*Drymodes brunneopygia*) and royal flycatcher (*Onychorhynchus coronatus*)) in relation to the exon patterns of *DNAJC15* in chicken, zebra finch and *L. aspasia* (Extended Data Fig. 7d).

### Selection analysis on whole-genome alignments

#### Neutral model

To estimate the degree of conservation or acceleration within a column requires evaluating the departure from a ‘neutral’ rate of evolution. This rate is described using a neutral model. We estimated a neutral model on the basis of ancestral repeats using an automatic pipeline (https://github.com/ComparativeGenomicsToolkit/neutral-model-estimator). We extracted the ancestral genome from the alignment representing the MRCA of all birds, and ran RepeatMasker^43^ to find avian repeats present in that genome (using the species library ‘aves’ from RepBase v.20170127^55^). A random sample of 100,000 bases within these repeats was used to extract a MAF, which was used as input to the phyloFit program from the PHAST v.1.5^56^ package to create the neutral model (using a general reversible model of nucleotide substitution). The PHAST framework allows only at most a single entry per genome per column, whereas the output MAFs may contain alignments to multiple copies. To resolve this, maf_stream (https://github.com/joelarmstrong/maf_stream) was used to combine multiple entries per genome into a single entry (using maf_stream dup_merge consensus).

Sex-determining chromosomes are known to evolve at a different rate than autosomes (the fast-X and fast-Z hypothesis)^10,29,57^. Furthermore, micro- and macro-chromosomes in birds have been shown to evolve at different rates as well^10,30^. To remove any potential differences in neutral nucleotide substitution rates among these chromosomes as a factor, we generated a second set of neutral models that represent the neutral rate on these three chromosome sets (we call this set the ‘three-rate model’). These models were generated by mapping the ancestral repeat sample from the root ancestral genome to the chicken genome, then separating the resulting bases into bins on the basis of whether they are in macro-, micro- and sex-chromosomes in chicken. For our purposes, we defined micro-chromosomes as any chicken autosomal chromosomes other than chromosomes 1–8. Then, we used the Cactus alignments and the chicken karyotypes to infer the chromosomal assignment for other species. The training was referenced on chicken, so we note that—owing to rare fusion or fission events—it is possible that some chicken micro-chromosomes may have become macro-chromosomes in other species or vice versa. We then scaled the ancestral-repeats model separately for each of the three bins using phyloFit --init-model <original model> --scale-only. This three-rate model was used for all selection-related results and figures in the Article by default, unless specifically mentioned otherwise.

#### Conservation and acceleration scores, and significance calls

**Extended Data Fig. 9:**
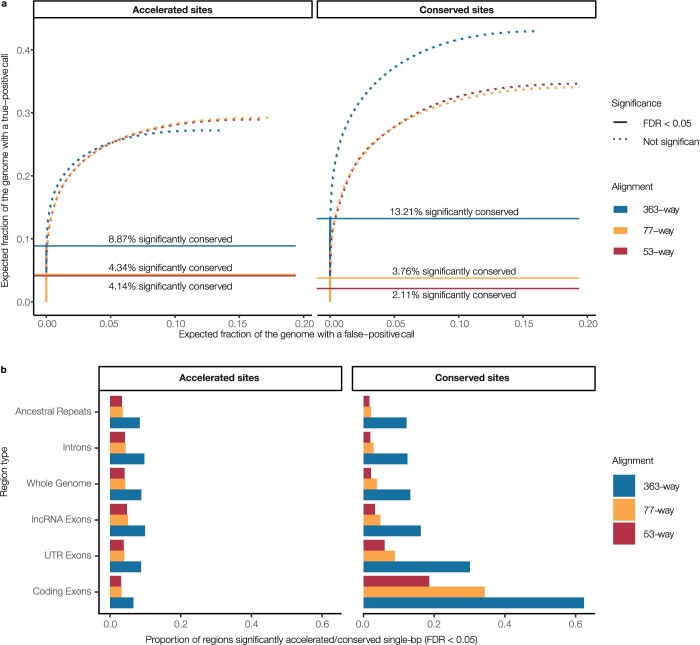
Acceleration and conservation scores. Results are shown from 3 alignments for 53 birds, 77 vertebrates, and 363 birds. **a**, Acceleration (left) and conservation (right) within alignment columns on chicken. This panel is similar to Fig. 3a, but includes accelerated columns. **b**, Proportion of chicken functional regions covered by significantly accelerated or conserved sites. This panel is similar to Fig. 3c, but includes accelerated columns.

We estimated conservation and acceleration scores for the B10K Project alignment using PhyloP^56,58^ run with the --method LRT and --mode CONACC scoring options. We ran this twice using the two neutral model sets described in ‘Neutral model’. When estimating the scores using the three-rate model we ran each chromosome separately, using the corresponding scaled model belonging to the proper set (macro-, micro- or sex-chromosomes). Although the HAL toolkit v.2.1 contains a tool that produces PhyloP scores, that tool works on the basis of alignment-wide columns, which combine all lineage-specific duplications into a single column: this is undesirable, as some alignment-wide columns containing homologies between two or more paralogues may be resolvable into multiple orthologous columns when viewed from chicken. Therefore, we instead ran PhyloP on a MAF export referenced on the chicken genome (using the hal2maf tool with the --onlyOrthologs option). These MAFs were post-processed using the maf_stream command. The results on acceleration and conservation scores are shown in Extended Data Fig. 9a.

We obtained the 77-way MULTIZ alignment from the UCSC Genome Browser^31^ (http://hgdownload.soe.ucsc.edu/goldenPath/galGal6/multiz77way/maf/). Rather than use the PhyloP scores provided by the browser (which were trained on fourfold-degenerate sites using a single neutral model), we estimated new scores using a three-rate model in the same manner as the 363-way alignment.

The 53-way scores were generated simply by providing the avian subtree of the 77-way tree (using the --tree option) when fitting the neutral model. Though the resulting scores are based on a different version of the chicken assembly than we used for the primary analysis (galGal6 instead of galGal4), most analyses did not need assembly coordinates. For one aspect of the analysis (the region-specific analysis in Extended Data Fig. 9b) we needed a common coordinate system, so we lifted these scores to galGal4 using the liftOver tool (16.2 Mb (1.5% of the total) were unable to be lifted over). The two score sets largely agreed on the direction of acceleration and conservation, with the values in the 363-way alignment being generally considerably higher owing to the additional power (Extended Data Fig. 9a).

PhyloP scores represent log-encoded *P* values of acceleration. We transformed these scores into *P* values, then into *q* values using the FDR-correcting method of Benjamini and Hochberg^33^. Any site that had a *q* value less than 0.05 was deemed significantly conserved or accelerated; Extended Data Fig. 9a provides the proportions of accelerated and conserved regions. We extracted the significantly accelerated and conserved sites from the PhyloP wiggle files using the Wiggletools v.1.2.3^59^ command wiggletools gt <threshold> abs, with the appropriate score threshold from Supplementary Table 15.

#### Intersection with functional regions of the genome

**Extended Data Fig. 10:**
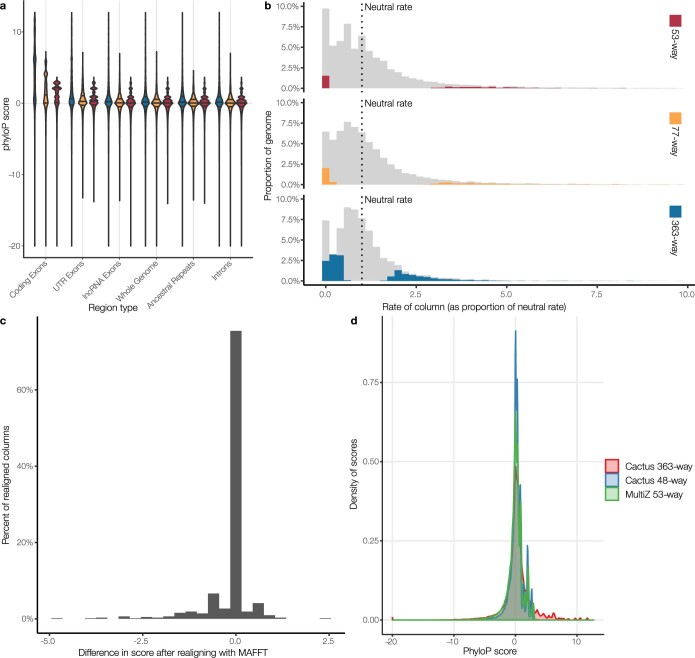
Distribution of acceleration and conservation scores. **a**, Distribution of conservation and acceleration scores within different functional region types across alignments. Lines mark quartiles of the density estimates. **b**, Larger histogram of chicken column rates. This panel is similar to Fig. 3b, but includes accelerated columns ending at a rate of 10× the neutral rate. **c**, Difference in PhyloP scores (compared to original scores) after realignment with MAFFT for a random sample of significantly conserved sites. **d**, Comparison of the distribution of PhyloP scores across alignments. Scores indicate log-scaled probabilities of conservation (positive values) or acceleration (negative values) for each base in the genome. **a** and **d** show results from three alignments for 53 birds, 77 vertebrates and 363 birds.

We split RefSeq genes (obtained via the RefSeq gene track on the galGal4 UCSC browser^31^) into sets of coding exons, UTR exons and introns. We also downloaded a lncRNA gene set from NONCODE v.5^60^ to obtain lncRNA regions and mapped all ancestral repeats from the root genome (as described in ‘Neutral model’) to chicken to get ancestral-repeat regions. All of these regions were made mutually exclusive by removing overlaps with all other region types. Finally, 100,000 bases were randomly sampled from each of these mutually exclusive regions and used to extract a corresponding distribution of scores for each region from the wiggle file. We identified transcription factor binding motifs on the basis of the chicken genome using JASPAR^61^. The results are shown in Fig. 3c, d, Extended Data Figs. 9b, 10a.

#### Distribution of rate of alignment columns

Finding the distribution of rates of alignment columns (relative to the neutral rate) is necessary for determining the strength of conservation that is needed for significance. We sampled 10,000 sites at random from each of the galGal4 (for the 363-way alignment) and galGal6 (for the 77-way alignment) assemblies. For the 363-way alignment, a MAF was exported containing the columns for each of these sites using hal2maf, and for the 77-way alignment the mafFrags program was used to obtain the columns from the UCSC browser database. The --base-by-base mode of PhyloP was used to obtain the ‘scale’ parameter for each column, which represents a best-fit multiplier of the neutral model applied to all branch lengths in the tree. For the 363-way alignment, we divided the columns within the MAF into three separate files according to their bin within the three-rate model, and used the appropriate model for each resulting MAF. The results are shown in Fig. 3b, Extended Data Fig. 10b.

#### Realignment of conserved sites

Our conservation and acceleration calls fundamentally rely on information from the alignment. For this reason, errors in the alignment could potentially cause erroneous acceleration or conservation calls. We tested the degree to which alignment choices for a given region affect our conservation calls. We sampled 1,000 sites randomly selected from the set of conserved sites in chicken and extracted their columns from the alignment. For each species in each column, we extracted a 2-kb region surrounding the aligned site into FASTA format, resulting in 1,000 FASTAs (one for each column). We then realigned these FASTAs using MAFFT^62^ and used PhyloP on the resulting region to extract a new score for the column containing the chicken site that was originally sampled.

#### Comparison to a 48-way alignment

We also constructed a 48-way Cactus alignment relating the 48 bird genomes used in phase I of the project. We then generated PhyloP scores on this 48-way alignment in the same manner as the other alignments described in ‘Conservation and acceleration scores, and significance calls’.

### Reporting summary

Further information on research design is available in the Nature Research Reporting Summary linked to this paper.

## Online content

Any methods, additional references, Nature Research reporting summaries, source data, extended data, supplementary information, acknowledgements, peer review information; details of author contributions and competing interests; and statements of data and code availability are available at 10.1038/s41586-020-2873-9.

## Supplementary information

Supplementary Information This file contains Supplementary Notes, Supplementary Methods and Supplementary Results regarding species selection, genome sequencing, assembly, annotation and ortholog identification, and whole-genome alignment. It also contains legends for Supplementary Tables 1-15. An interactive supplementary figure is available at https://genome-b10k.herokuapp.com/main. An interactive plot of assembly statistics and annotation statistics for all 363 bird genomes, data can be shown by species, taxonomy or by the source of the genome sequence. This figure visualises data from Supplementary Table 1.

Reporting Summary

Supplementary Data The tree file in newick format for all 10,135 species of birds. The tree was pruned from the synthesis tree by excluding all subspecies, operational taxonomic units and unaccepted species as described in the Supplementary Information. Also available on Mendeley Data (doi:10.17632/fnpwzj37gw).

Supplementary Tables This file contains Supplementary Tables 1-15 – see Supplementary Information document for legends. Also available on Mendeley Data (doi:10.17632/fnpwzj37gw). Sample information for each genome and genome statistics (Supplementary Table 1) can also be viewed online at https://b10k.scifeon.cloud/.

## Data Availability

All data released with this Article can be freely used. The B10K consortium is organizing phylogenomic analyses and other analyses with the whole-genome alignment, and we encourage persons to contact us for collaboration. Genome sequencing data, the genome assemblies and annotations of 267 species generated in this study have been deposited in the NCBI SRA and GenBank under accession PRJNA545868. The above data have also been deposited in the CNSA (https://db.cngb.org/cnsa/) of CNGBdb with accession number CNP0000505. The mitochondrial genomes and annotations of 336 species have been deposited in the NCBI GenBank under PRJNA545868. Sample information for each genome and the genome statistics can also be viewed online at https://b10k.scifeon.cloud/. The whole-genome alignment of the 363 birds in HAL format, along with a UCSC browser hub for all 363 species, is available at https://cglgenomics.ucsc.edu/data/cactus/. The Supplementary Data, which contains the tree file in Newick format for all 10,135 species of birds, is also available on Mendeley Data (10.17632/fnpwzj37gw). The tree was pruned from the synthesis tree by excluding all subspecies, operational taxonomic units and unaccepted species as described in the Supplementary Information. Other data generated and analysed during this study, including Supplementary Tables 1–15, are also available on Mendeley Data (10.17632/fnpwzj37gw). The study used publicly available data for species confirmation from the Barcode of Life Data (BOLD) (http://www.barcodinglife.org) and NCBI (https://www.ncbi.nlm.nih.gov/). The reference genomes, gene sets and published RNA-sequencing data used in the gene annotation and alignment construction of this study are available from Ensembl (http://www.ensembl.org) and NCBI. The databases used in functional annotation are available in InterPro (https://www.ebi.ac.uk/interpro), SwissProt (https://www.uniprot.org) and KEGG (https://www.genome.jp/kegg). The database used in the transposable elements annotation is available online (http://www.repeatmasker.org). The 77-way MULTIZ alignment, RefSeq genes and lncRNA gene set used in the selection analysis is available in UCSC Genome Browser (http://www.genome.ucsc.edu) and NONCODEv.5 database (http://www.noncode.org). The JASPAR2020 CORE vertebrate database used to identify transcription factor binding motifs is available online (http://jaspar2020.genereg.net).

## Code availability

Scripts to run the annotation pipeline and the orthologue assignment pipeline can be found on the B10K GitHub repository at https://github.com/B10KGenomes/annotation. Scripts to estimate the neutral model can be found at https://github.com/ComparativeGenomicsToolkit/neutral-model-estimator.
