# Author Correction: Dense sampling of bird diversity increases power of comparative genomics

**DOI:** 10.1038/s41586-021-03473-8

**Published:** 2021-04-08

**Authors:** Shaohong Feng, Josefin Stiller, Yuan Deng, Joel Armstrong, Qi Fang, Andrew Hart Reeve, Duo Xie, Guangji Chen, Chunxue Guo, Brant C. Faircloth, Bent Petersen, Zongji Wang, Qi Zhou, Mark Diekhans, Wanjun Chen, Sergio Andreu-Sánchez, Ashot Margaryan, Jason Travis Howard, Carole Parent, George Pacheco, Mikkel-Holger S. Sinding, Lara Puetz, Emily Cavill, Ângela M. Ribeiro, Leopold Eckhart, Jon Fjeldså, Peter A. Hosner, Robb T. Brumfield, Les Christidis, Mads F. Bertelsen, Thomas Sicheritz-Ponten, Dieter Thomas Tietze, Bruce C. Robertson, Gang Song, Gerald Borgia, Santiago Claramunt, Irby J. Lovette, Saul J. Cowen, Peter Njoroge, John Philip Dumbacher, Oliver A. Ryder, Jérôme Fuchs, Michael Bunce, David W. Burt, Joel Cracraft, Guanliang Meng, Shannon J. Hackett, Peter G. Ryan, Knud Andreas Jønsson, Ian G. Jamieson, Rute R. da Fonseca, Edward L. Braun, Peter Houde, Siavash Mirarab, Alexander Suh, Bengt Hansson, Suvi Ponnikas, Hanna Sigeman, Martin Stervander, Paul B. Frandsen, Henriette van der Zwan, Rencia van der Sluis, Carina Visser, Christopher N. Balakrishnan, Andrew G. Clark, John W. Fitzpatrick, Reed Bowman, Nancy Chen, Alison Cloutier, Timothy B. Sackton, Scott V. Edwards, Dustin J. Foote, Subir B. Shakya, Frederick H. Sheldon, Alain Vignal, André E. R. Soares, Beth Shapiro, Jacob González-Solís, Joan Ferrer-Obiol, Julio Rozas, Marta Riutort, Anna Tigano, Vicki Friesen, Love Dalén, Araxi O. Urrutia, Tamás Székely, Yang Liu, Michael G. Campana, André Corvelo, Robert C. Fleischer, Kim M. Rutherford, Neil J. Gemmell, Nicolas Dussex, Henrik Mouritsen, Nadine Thiele, Kira Delmore, Miriam Liedvogel, Andre Franke, Marc P. Hoeppner, Oliver Krone, Adam M. Fudickar, Borja Milá, Ellen D. Ketterson, Andrew Eric Fidler, Guillermo Friis, Ángela M. Parody-Merino, Phil F. Battley, Murray P. Cox, Nicholas Costa Barroso Lima, Francisco Prosdocimi, Thomas Lee Parchman, Barney A. Schlinger, Bette A. Loiselle, John G. Blake, Haw Chuan Lim, Lainy B. Day, Matthew J. Fuxjager, Maude W. Baldwin, Michael J. Braun, Morgan Wirthlin, Rebecca B. Dikow, T. Brandt Ryder, Glauco Camenisch, Lukas F. Keller, Jeffrey M. DaCosta, Mark E. Hauber, Matthew I. M. Louder, Christopher C. Witt, Jimmy A. McGuire, Joann Mudge, Libby C. Megna, Matthew D. Carling, Biao Wang, Scott A. Taylor, Glaucia Del-Rio, Alexandre Aleixo, Ana Tereza Ribeiro Vasconcelos, Claudio V. Mello, Jason T. Weir, David Haussler, Qiye Li, Huanming Yang, Jian Wang, Fumin Lei, Carsten Rahbek, M. Thomas P. Gilbert, Gary R. Graves, Erich D. Jarvis, Benedict Paten, Guojie Zhang

**Affiliations:** 1 China National GeneBank, BGI-Shenzhen, Shenzhen, China; 2 State Key Laboratory of Genetic Resources and Evolution, Kunming Institute of Zoology, Chinese Academy of Sciences, Kunming, China; 3 BGI-Shenzhen, Shenzhen, China; 4 Villum Centre for Biodiversity Genomics, Section for Ecology and Evolution, Department of Biology, University of Copenhagen, Copenhagen, Denmark; 5 UC Santa Cruz Genomics Institute, UC Santa Cruz, Santa Cruz, CA USA; 6 Natural History Museum of Denmark, University of Copenhagen, Copenhagen, Denmark; 7 BGI Education Center, University of Chinese Academy of Sciences, Shenzhen, China; 8 Department of Biological Sciences, Louisiana State University, Baton Rouge, LA USA; 9 Museum of Natural Science, Louisiana State University, Baton Rouge, LA USA; 10 Centre of Excellence for Omics-Driven Computational Biodiscovery (COMBio), Faculty of Applied Sciences, AIMST University, Kedah, Malaysia; 11 Section for Evolutionary Genomics, The GLOBE Institute, Faculty of Health and Medical Sciences, University of Copenhagen, Copenhagen, Denmark; 12 MOE Laboratory of Biosystems Homeostasis and Protection, Life Sciences Institute, Zhejiang University, Hangzhou, China; 13 Department of Neuroscience and Developmental Biology, University of Vienna, Vienna, Austria; 14 Center for Reproductive Medicine, The 2nd Affiliated Hospital, School of Medicine, Zhejiang University, Hangzhou, China; 15 Institute of Molecular Biology, National Academy of Sciences, Yerevan, Armenia; 16 Novogene, Durham, NC USA; 17 Duke University Medical Center, Durham, NC USA; 18 Department of Dermatology, Medical University of Vienna, Vienna, Austria; 19 Center for Macroecology, Evolution, and Climate, GLOBE Institute, University of Copenhagen, Copenhagen, Denmark; 20 Southern Cross University, Coffs Harbour, New South Wales Australia; 21 Centre for Zoo and Wild Animal Health, Copenhagen Zoo, Frederiksberg, Denmark; 22 Center of Natural History, Universität Hamburg, Hamburg, Germany; 23 Department of Zoology, University of Otago, Dunedin, New Zealand; 24 Key Laboratory of Zoological Systematics and Evolution, Institute of Zoology, Chinese Academy of Sciences, Beijing, China; 25 Environmental Futures Research Institute, Griffith University, Nathan, Queensland Australia; 26 Department of Biology, University of Maryland, College Park, MD USA; 27 Department of Natural History, Royal Ontario Museum, Toronto, Ontario Canada; 28 Department of Ecology and Evolutionary Biology, University of Toronto, Toronto, Ontario Canada; 29 Cornell Lab of Ornithology, Cornell University, Ithaca, NY USA; 30 Biodiversity and Conservation Science, Department of Biodiversity Conservation and Attractions, Perth, Western Australia Australia; 31 Ornithology Section, Zoology Department, National Museums of Kenya, Nairobi, Kenya; 32 Ornithology and Mammalogy, California Academy of Sciences, San Francisco, CA USA; 33 San Diego Zoo Institute for Conservation Research, Escondido, CA USA; 34 Evolution, Behavior, and Ecology, Division of Biology, University of California San Diego, La Jolla, CA USA; 35 Institut de Systématique, Evolution, Biodiversité (ISYEB), Muséum National d’Histoire Naturelle, CNRS, Sorbonne Université, EPHE, Université des Antilles, Paris, France; 36 Trace and Environmental DNA (TrEnD) Laboratory, School of Molecular and Life Sciences, Curtin University, Western Australia, Perth, Australia; 37 UQ Genomics, University of Queensland, Brisbane, Queensland Australia; 38 Department of Ornithology, American Museum of Natural History, New York, NY USA; 39 Integrative Research Center, Field Museum of Natural History, Chicago, IL USA; 40 FitzPatrick Institute of African Ornithology, University of Cape Town, Cape Town, South Africa; 41 Department of Biology, University of Florida, Gainesville, FL USA; 42 Department of Biology, New Mexico State University, Las Cruces, NM USA; 43 Department of Electrical and Computer Engineering, University of California San Diego, La Jolla, CA USA; 44 Department of Ecology and Genetics – Evolutionary Biology, Evolutionary Biology Centre (EBC), Science for Life Laboratory, Uppsala University, Uppsala, Sweden; 45 Department of Organismal Biology – Systematic Biology, Evolutionary Biology Centre (EBC), Science for Life Laboratory, Uppsala University, Uppsala, Sweden; 46 School of Biological Sciences, University of East Anglia, Norwich, UK; 47 Department of Biology, Lund University, Lund, Sweden; 48 Institute of Ecology and Evolution, University of Oregon, Eugene, OR USA; 49 Department of Plant and Wildlife Sciences, Brigham Young University, Provo, UT USA; 50 Data Science Lab, Office of the Chief Information Officer, Smithsonian Institution, Washington, DC USA; 51 Focus Area for Human Metabolomics, North-West University, Potchefstroom, South Africa; 52 Department of Animal Sciences, University of Pretoria, Pretoria, South Africa; 53 Department of Biology, East Carolina University, Greenville, NC USA; 54 Department of Molecular Biology and Genetics, Cornell University, Ithaca, NY USA; 55 Avian Ecology Program, Archbold Biological Station, Venus, FL USA; 56 Department of Biology, University of Rochester, Rochester, NY USA; 57 Department of Organismic and Evolutionary Biology, Harvard University, Cambridge, MA USA; 58 Museum of Comparative Zoology, Harvard University, Cambridge, MA USA; 59 Informatics Group, Harvard University, Cambridge, MA USA; 60 Sylvan Heights Bird Park, Scotland Neck, NC USA; 61 GenPhySE, INRA, INPT, INP-ENVT, Université de Toulouse, Castanet-Tolosan, France; 62 Laboratório Nacional de Computação Científica, Petrópolis, Brazil; 63 Department of Ecology and Evolutionary Biology, University of California Santa Cruz, Santa Cruz, CA USA; 64 Howard Hughes Medical Institute, University of California Santa Cruz, Santa Cruz, CA USA; 65 Institut de Recerca de la Biodiversitat (IRBio), Universitat de Barcelona, Barcelona, Spain; 66 Departament de Biologia Evolutiva, Ecologia i Ciències Ambientals (BEECA), Universitat de Barcelona, Barcelona, Spain; 67 Departament de Genètica, Microbiologia i Estadística, Universitat de Barcelona, Barcelona, Spain; 68 Department of Molecular, Cellular and Biomedical Sciences, University of New Hampshire, Durham, NH USA; 69 Department of Biology, Queen’s University, Kingston, Ontario Canada; 70 Department of Bioinformatics and Genetics, Swedish Museum of Natural History, Stockholm, Sweden; 71 Centre for Palaeogenetics, Stockholm, Sweden; 72 Milner Centre for Evolution, University of Bath, Bath, UK; 73 Instituto de Ecologia, UNAM, Mexico City, Mexico; 74 State Key Laboratory of Biocontrol, School of Ecology, Sun Yat-sen University, Guangzhou, China; 75 Center for Conservation Genomics, Smithsonian Conservation Biology Institute, Smithsonian Institution, Washington, DC USA; 76 New York Genome Center, New York, NY USA; 77 Department of Anatomy, University of Otago, Dunedin, New Zealand; 78 AG Neurosensory Sciences, Institut für Biologie und Umweltwissenschaften, University of Oldenburg, Oldenburg, Germany; 79 Biology Department, Texas A&M University, College Station, TX USA; 80 MPRG Behavioural Genomics, Max Planck Institute for Evolutionary Biology, Plön, Germany; 81 Institute of Clinical Molecular Biology, Christian-Albrechts- University of Kiel, Kiel, Germany; 82 Department of Wildlife Diseases, Leibniz Institute for Zoo and Wildlife Research, Berlin, Germany; 83 Environmental Resilience Institute, Indiana University, Bloomington, IN USA; 84 National Museum of Natural Sciences, Spanish National Research Council (CSIC), Madrid, Spain; 85 Department of Biology, Indiana University, Bloomington, IN USA; 86 Institute of Marine Science, University of Auckland, Auckland, New Zealand; 87 Center for Genomics and Systems Biology, Department of Biology, New York University – Abu Dhabi, Abu Dhabi, UAE; 88 Wildlife and Ecology Group, Massey University, Palmerston North, New Zealand; 89 School of Fundamental Sciences, Massey University, Palmerston North, New Zealand; 90 Departamento de Bioquímica e Biologia Molecular, Centro de Ciências, Universidade Federal do Ceará, Fortaleza, Brazil; 91 Laboratório de Genômica e Biodiversidade, Instituto de Bioquímica Médica Leopoldo de Meis, Rio de Janeiro, Brazil; 92 Department of Biology, University of Nevada Reno, Reno, NV USA; 93 Department of Integrative Biology and Physiology, UCLA, Los Angeles, CA USA; 94 Smithsonian Tropical Research Institute, Panama City, Panama; 95 Department of Wildlife Ecology and Conservation, University of Florida, Gainesville, FL USA; 96 Center for Latin American Studies, University of Florida, Gainesville, FL USA; 97 Department of Biology, George Mason University, Fairfax, VA USA; 98 Department of Biology and Neuroscience Minor, University of Mississippi, University, MS USA; 99 Department of Ecology and Evolutionary Biology, Brown University, Providence, RI USA; 100 Max Planck Institute for Ornithology, Seewiesen, Germany; 101 Department of Vertebrate Zoology, National Museum of Natural History, Smithsonian Institution, Washington, DC USA; 102 Behavior, Ecology, Evolution and Systematics Program, University of Maryland, College Park, MD USA; 103 Computational Biology Department, Carnegie Mellon University, Pittsburgh, PA USA; 104 Migratory Bird Center, Smithsonian National Zoological Park and Conservation Biology Institute, Washington, DC USA; 105 Department of Evolutionary Biology and Environmental Studies, University of Zurich, Zurich, Switzerland; 106 Biology Department, Boston College, Chestnut Hill, MA USA; 107 Department of Evolution, Ecology, and Behavior, School of Integrative Biology, University of Illinois at Urbana-Champaign, Urbana, IL USA; 108 International Research Center for Neurointelligence, University of Tokyo, Tokyo, Japan; 109 Museum of Southwestern Biology, Department of Biology, University of New Mexico, Albuquerque, NM USA; 110 Museum of Vertebrate Zoology, Department of Integrative Biology, University of California, Berkeley, Berkeley, CA USA; 111 National Center for Genome Resources, Santa Fe, NM USA; 112 Department of Zoology and Physiology, University of Wyoming, Laramie, WY USA; 113 School of BioSciences, The University of Melbourne, Melbourne, Victoria Australia; 114 Department of Ecology and Evolutionary Biology, University of Colorado Boulder, Boulder, CO USA; 115 Finnish Museum of Natural History, University of Helsinki, Helsinki, Finland; 116 Department of Behavioral Neuroscience, Oregon Health and Science University, Portland, OR USA; 117 Department of Biological Sciences, University of Toronto Scarborough, Toronto, Ontario Canada; 118 James D. Watson Institute of Genome Sciences, Hangzhou, China; 119 Center for Excellence in Animal Evolution and Genetics, Chinese Academy of Sciences, Kunming, China; 120 Danish Institute for Advanced Study, University of Southern Denmark, Odense, Denmark; 121 Institute of Ecology, Peking University, Beijing, China; 122 Department of Life Sciences, Imperial College London, Ascot, UK; 123 University Museum, Norwegian University of Science and Technology, Trondheim, Norway; 124 The Rockefeller University, New York, NY USA; 125 Howard Hughes Medical Institute, Chevy Chase, MD USA

**Keywords:** Evolutionary genetics, Comparative genomics

Correction to: *Nature* 10.1038/s41586-020-2873-9 Published online 11 November 2020

In Supplementary Table 1 of this Article, 23 samples (B10K-DU-029-32, B10K-DU-029-33, B10K-DU-029-36 to B10K-DU-029-44, B10K-DU-029-46, B10K-DU-029-47, B10K-DU-029-49 to B10K-DU-029-53, B10K-DU-029-75 to B10K-DU-029-77, B10K-DU-029-80, and B10K-DU-030-03; styled in boldface in the revised table) were assigned to the incorrect institution. Supplementary Table 1 has been amended to reflect the correct source institution for these samples, and associated data (tissue, museum ID/source specimen ID, site, state/province, latitude, longitude, date collected and sex) have been updated accordingly. The original table is provided as Supplementary Information to this Amendment, and the original Article has been corrected online.

**Supplementary information** is available in the online version of this Amendment.

## Supplementary information

Supplementary Information This file contains the original incorrect Supplementary Table 1.

