# Supplementary material for: Dense sampling of bird diversity increases power of comparative genomics
Source: Nature. 2020 Nov 11;587(7833):252–7. doi: 10.1038/s41586-020-2873-9 (PMC7759463; doi:10.1038/s41586-020-2873-9)
Supplement: Supplementary file 1 — This file contains Supplementary Notes, Supplementary Methods and Supplementary Results regarding species selection, genome sequencing, assembly, annotation and ortholog identification, and whole-genome alignment. It also contains legends for Supplementary Tables 1-15. An interactive supplementary figure is available at https://genome-b10k.herokuapp.com/main. An interactive plot of assembly statistics and annotation statistics for all 363 bird genomes, data can be shown by species, taxonomy or by the source of the genome sequence. This figure visualises data from Supplementary Table 1. [file 41586_2020_2873_MOESM1_ESM.pdf]

natureresearch

https://doi.org/10.1038/s41586-020-2873-9

Supplementary information

Dense sampling of bird diversity increases power of comparative genomics

In the format provided by the authors and unedited

Supplementary Information

Dense sampling of bird diversity increases power of comparative genomics ---- Supplementary File 1

Supplementary Notes
- Supplementary acknowledgements

Supplementary Methods
- Data summary
- Sample selection
  - Families represented and missing from the sampling
  - Taxonomy used
  - Phylogenetic coverage of the genomes
- Extraction of genomic DNA
- Library construction & sequencing
- Assembly
  - Assembly strategy using SOAPdenovo
  - Assembly strategy using Allpaths-LG
  - Assembly strategies for external (OUT) genomes
- Genome assembly quality assessment
- Genome completeness
- DNA barcoding for quality control and species confirmation
- Mitochondrial genome assembly and annotation
- Protein coding genes annotation
  - Primary reference gene set.
  - Supplemental human gene set.
  - Supplemental transcriptome gene set.
- Repeat annotation
- Cactus whole-genome alignment
- Intron dataset construction
- Codon preference
- Analyses of gene duplication, gene loss and pseudogenes
  - Detecting gene loss in avian genomes
  - Rhodopsin/opsins and vision
  - Growth hormone (*GH*) duplication in Passeriformes
  - Loss of Cornulin (*CRNN*) in songbirds

Supplementary Results
- Genome assembly quality

Genome completeness
Mitochondrial genomes
Repeat content
Cactus whole-genome alignment
Ortholog identification
Dataset sizes for different genomic categories
GC content
Codon preference
Analyses of gene duplication, gene loss and pseudogenes
Detecting gene loss in avian genomes
Rhodopsin/opsins and vision
Growth hormone (*GH*) duplication in Passeriformes
Loss of Cornulin (*CRNN*) in songbirds
Lineage-specific sequences based on whole-genome alignments
Selection analysis on whole-genome alignments
Realignment of conserved sites
Comparison to a 48-way alignment

Supplementary Tables

References

Supplementary Notes

Supplementary acknowledgements

K.A.J. thanks the Carlsberg Foundation (CF15–0079, CF17-0248) and the Villum Foundation (15560) for supporting field efforts, and Vojtěch Novotný at the Binatang Research Center, Bulisa Iova at the PNG National Museum and Art Gallery and a number of local communities for facilitating fieldwork in Papua New Guinea. G.Ca. and L.F.K. thank Pirmin Nietlisbach for helping with sample collection, Lucy Poveda and Andrea Patrignani (Functional Genomics Center Zurich) for support with library preparation and sequencing and Heidi Tschanz-Lischer for advice on assembly. Q.Z. is supported by National Natural Science Foundation of China (grant nos. 31671319,31722050, 32061130208), Natural Science Foundation of Zhejiang Province (LD19C190001), and European Research Council Starting Grant (grant agreement 677696). H.M. and M.L. acknowledge funding from SFB 1372 (Deutsche Forschungsgemeinschaft).

Funding for individual genomes was provided by the following sources:

- *Alca torda, Copsychus sechellarum, Cercotrichas coryphoeus:* ERC Consolidator grant 681396 (M.T.P.G.)
- *Vidua chalybeata, Molothrus ater*: US NSF 1456612 (C.N.B.) & 1456524 (M.E.H.)
- *Asarcornis scutulata*: Akron Zoo (C.N.B.), International Wild Waterfowl Trust (C.N.B.), Carolinas/Virginia Pheasant and Waterfowl Society (C.N.B.)
- *Manacus manacus:* US NSF 1457541 (B.A.L.)
- *Steatornis caripensis:* SISBIO-Aves project funded by CNPq/FAPESPA, Brazil (A.T.R.V., C.V.M., A.A.)
- *Acrocephalus arundinaceus*, *Atlantisia rogersi*, *Cettia cetti*, *Nesospiza acunhae*: Swedish Research Council, 621-2014-5222 and 621-2016-689 (B.H.)
- *Certhia familiaris*: SciLifeLab Swedish Biodiversity Program, 2015-R14 (A.S.). A.S. acknowledges support from the National Genomics Infrastructure in Stockholm funded by Science for Life Laboratory, the Knut and Alice Wallenberg Foundation and the Swedish Research Council, and SNIC/Uppsala Multidisciplinary Center for Advanced Computational Science for assistance with massively parallel sequencing and access to the UPPMAX computational infrastructure.

Supplementary Methods

Data summary

For Phase II of the B10K (the “family phase”), we included a total of 363 species from 218 families. The 363 genomes came from four data sources which included 268 newly

sequenced genomes and 95 publicly available genomes (Extended Data Fig. 1a, Supplementary Table 1):

1. B10K genomes: 236 species newly sequenced for this phase (prefixed with "B10K"), of which one has been previously released to NCBI[1], the remaining 235 species are released here;
2. OUT genomes: 49 species provided by other research groups (prefixed with "OUT"). OUT genomes provided by individual labs were unpublished when they were integrated into the B10K analyses. 17 have since been released to NCBI and 32 others are being made public here alongside the B10K genomes (Supplementary Table S2);
3. Avian Phylogenomics Project (APP) genomes: 42 publicly available genomes from Phase I of the B10K (prefixed with "APP")[2,3];
4. NCBI genomes: 36 species from the genome database of NCBI (prefixed with "NCBI").

Publicly available NCBI genomes and OUT genomes from other labs were only considered if they passed the assembly quality criteria of contig N50 > 5 kb, scaffold N50 > 30 kb and total assembly length > 0.9 Gb (average bird genome is ~1.2 Gb). The processing steps described in the following sections first refer to the B10K genomes. The OUT genomes that are released here were generated with a variety of methods, which will be summarised at the end of each section.

236 B10K genomes remained from a total 272 sequenced species after excluding the following samples (summarised in Extended Data Fig. 1c, Supplementary Table 6):

1. 13 genomes were removed due to poor genome assembly quality (scaffold N50 < 10 kb and/or total assembly length < 0.9 Gb), with details in section Genome assembly quality assessment (Supplementary Table 3);
2. 13 genomes were removed because of potential contamination of the sample, with details in the section DNA barcoding for quality control and species confirmation (Supplementary Table 5);
3. 5 species misidentifications were corrected, with details given in the DNA barcoding section for quality control and species confirmation;
4. 10 genomes were redundant with a genome of better quality available in NCBI or from external labs (OUT genomes);

<u>Sample selection</u>

A total of 236 genomes were sequenced specifically for this project, drawing on samples from 17 scientific collections. Museums listed in Supplementary Table 1 issued written

permission to sequence, analyze, and publish the genetic material provided by them to the B10K consortium. The three largest contributing institutes were the National Museum of Natural History of the Smithsonian Institution (140 species), Louisiana State University Museum of Natural Science (31 species) and Southern Cross University (23 species). We preferentially chose samples from wild-caught individuals with museum specimen vouchers.

A total of 42 of the 45 genomes sequenced by the Avian Phylogenomics Project[2–4] were used here, the remaining three had improved or updated genome releases available. Another 36 genomes were publicly available from NCBI. One B10K genome has already been made available (B10K-DU-002-22, Raggiana Bird-of-paradise *Paradisaea raggiana*[1]). The B10K ID is composed of different parts, the institution at which the library was prepared (DU for Duke University; IZ for Institute of Zoology, CAS; CU and UC for University of Copenhagen), and the row and column location in the freezer storage box the sample resides. B10K-DU-002-22 for example was prepared at Duke University and the sample is stored in row 002 and column 22 of the storage box.

In 2017, a call for unpublished bird genomes to be included in the B10K dataset was posted on the Evolution Directory EvolDir (http://life.biology.mcmaster.ca/~brian/evoldir.html). Of the responses, a total of 49 genomes passed the assembly quality criteria and were included in Phase II of the B10K. Of these 49, 17 have been released since, while the remaining 32 are being made available alongside the B10K genomes. Together, this adds to 267 newly released genomes.

Specimen collection data for the 363 sampled species can be accessed on the B10K website (https://b10k.genomics.cn/species.html). The specimens were sampled worldwide from every continent (Extended Data Fig. 1b). The IUCN RedList assesses 68 of the included species (19%) in the categories of concern: 2 Critically Endangered, 12 Endangered, 27 Vulnerable, 27 Near Threatened (IUCN, accessed June 2019) (Supplementary Table 1).

Families represented and missing from the sampling

We identified 236 extant bird families for potential inclusion in the analysis based on Howard & Moore 4th edition[5]. The 363 genomes fall into 218 of these families (92.4%). This is more than three times the taxonomic coverage encompassed by currently available genomes (63 families). Species representatives from additional deep branches of non-passerine families were included to more densely sample parts of the avian family tree with uncertain topology.

A total of 18 families were missing. For 10 families, no samples appropriate for genomic sequencing were available: Calyptophilidae, Hyliotidae, Melampittidae, Melanopareiidae, Mitrospingidae, Mohoidae, Phaenicophilidae, Pityriasidae, Psophodidae, and Zeledoniidae. The lack of suitable tissue samples was mostly due to difficulties in

obtaining collection permits for certain regions, or because the relevant species are rare or otherwise difficult to collect in the field. Tissue from a few missing families was available in collections but yielded poor DNA quality. Mohoidae have been extinct since the 1980s. Three other families are not represented in the final dataset because the sequenced genomes did not pass genome assembly quality control (see details in section Genome assembly quality assessment, Supplementary Table 3), namely Aegithinidae, Pluvianidae and Sarothruridae. Five families are not represented in the final dataset because the sequenced genomes were suspected to have been contaminated or mislabeled (see details in section DNA barcoding for quality control and species confirmation, Supplementary Table 3 and 5), namely Conopophagidae, Dulidae, Hypocoliidae, Pnoepygidae, and Stenostiridae.

The majority of families are represented by a single species (143 families, 65.6%) but Tinamidae has 10 species sequenced, Cuculidae has 7, and Muscicapidae has 6. For 28 families, all species have already been sequenced, which is largely due to 26 monotypic families, in addition to 2 families with 2 species (Cariamidae, Rheidae).

Taxonomy used

The species names follow the taxonomy of the online version of Howard & Moore 4th edition[5]. This produces some incongruences with the names used for published genomes in NCBI or in other taxonomic systems and the names we use here. Specifically,

- Northern Brown Kiwi (*Apteryx mantelli*, NCBI-005), a genome available on NCBI, is treated as *A. australis mantelli* on NCBI. *Apteryx mantelli* is treated as a full species in[5]. *Apteryx australis* is the Southern Brown Kiwi, while the original publication indicates that the sampled taxon was a Northern Brown Kiwi (*A. mantelli*) from the North Island of New Zealand[6].
- Karoo Scrub Robin (*Cercotrichas coryphoeus*, OUT-0024) is sometimes spelled *coryphaeus* in other sources (e.g. AviBase).
- Carrion Crow (*Corvus corone*, NCBI-020), a genome available on NCBI, is treated as *C. cornix cornix* on NCBI. *Corvus cornix* is not accepted as a species by[5] but as a subspecies (*C. corone cornix*).
- Red Crossbill (*Loxia curvirostra*, OUT-0011), is accepted by some taxonomic authorities as a separate species (*L. sinesciuris*) but not by[5].
- Bearded Manakin (*Manacus manacus*, OUT-0047), a genome available on NCBI, is treated as *M. vitellinus* on NCBI. *Manacus vitellinus* is treated not as a species but as a subspecies (*M. manacus vitellinus*)[5].

Phylogenetic coverage of the genomes

In order to visualise the distribution of genomic resources for all bird species (Fig. 1), we used the latest mega-phylogeny by Brown et al. (2017)[7] to highlight species with genomic information on the bird tree of life. This mega-phylogeny is a synthesis of published phylogenies and taxonomic information rather than a direct phylogenetic analysis. It contains 13,579 taxonomic units, more than the 10,135 species in Howard & Moore 4th edition[5], which stems from the inclusion of subspecies and operational names as separate tree terminals. For our purposes of highlighting phylogenetic coverage of high-level groups, those additional taxonomic units were not useful.

We took a number of steps to match tree terminals with the 10,135 species recognised by Howard & Moore 4th edition[5]. First, we collapsed all subspecies into one terminal, which left 11,709 species in the tree. Second, 1,102 species in 474 genera that were present in the Howard & Moore checklist but not in the tree (due to different taxonomic systems) were added to the tree, based on taxonomic information. If a missing species had different members of its genus in the tree, as was the case for species in 237 genera, we attached the missing species to the most recent common ancestor (MRCA) of its genus. If only one member of the genus was in the tree (and hence there was no MRCA node of the genus), the missing species was attached one node down. This was the case for species in 58 genera. For 179 genera, the genus was not represented in the tree at all. These species were attached at the node corresponding to the MRCA of other members of the family. Afterwards, we removed all terminals that were present in the tree but not recognised by the Howard & Moore checklist (2,676 species). The remaining 10,135 terminals in the final tree (Supplementary File 2) are the species recognised by Howard & Moore. The tree was rooted on Palaeognathae and species with available genomes were highlighted using ggtree (v2.2.1)[8].

Extraction of genomic DNA

In order to recover genomic DNA of appropriate quality and concentration, different extractions were performed depending on the tissue type. DNA was extracted using commercial extraction kits following the manufacturers' guidelines yielding high molecular weight DNA, even for the oldest sequenced samples. Blood samples were processed using the DNeasy Blood & Tissue Kit (Qiagen, Valencia-CA, USA), while tissue samples were processed using the KingFisher Cell and Tissue DNA Kit (Thermo Fisher 97030196) in combination with the KingFisher Duo Prime Purification System (Thermo Fisher Scientific, Waltham, MA, USA). Resulting DNA extracts were quantified using the Qubit 2.0 Fluorometer (Thermo Fisher Scientific, Waltham, MA, USA) with the standard protocol. To

check molecular integrity, each DNA extract was run on the 2200 TapeStation (Agilent Technologies, Santa Clara, CA, USA) following the manufacturer's protocol.

DNA extraction for the OUT genomes from external laboratories was done with different techniques using kits or commercial services or standard extractions for genomic DNA (Supplementary Table 2).

Library construction & sequencing

B10K genomes were sequenced at BGI using the Illumina HiSeq platforms. For most samples, pair-end libraries of one or two small insert sizes (250 bp, 280 bp, 500 bp, 800 bp) and one mate pair library (2 kb) were constructed. For the North Island Kokako (*Callaeas wilsoni*) and the Noisy Scrubbird (*Atrichornis clamosus*), we only had libraries for two small insert sizes (250 bp, 800 bp) due to limited genomic samples. The read length for small insert libraries was 150 bp, and 49 bp for the mate pair library. The sequencing depth for most avian genomes ranged from 35x to 123x (Supplementary Table 1).

OUT genomes and NCBI genomes were sequenced with a variety of sequencing technologies of mostly Illumina short read sequencing and a few PacBio SMRT sequencing (Supplementary Table 2).

Assembly

For the samples sequenced at BGI, we used SOAPdenovo v2.04[9] for all 272 samples and Allpaths-LG (v52488)[10] for the 99 samples that had an overlapping library (250 bp) and the mate pair library. For samples assembled with both methods, we selected the version with the higher quality based on the scaffold N50 and contig N50 values. Of the 99 samples, 74 had better assemblies with the Allpaths-LG assembly approach.

Assembly strategy using SOAPdenovo

Quality control steps on the raw reads prior to assembly were

1. Removing reads with more than 10% of N bases;
2. Removing reads with more than 40% low quality bases (Phred score ≤ 7);
3. Removing reads with undersize insert size;
4. Filtering out the PCR duplicates (if read1 and read2 of the same paired-end reads were identical).

After filtering the raw reads, the small insert size library data was split into an appropriate K-mer size to construct a *de Bruijn* graph. The graph was simplified by merging K-mer clipping tips, merging bubbles and removing low coverage links. All qualified data with unambiguous

connections in the *de Bruijn* graph were connected into contig sequences. All filtered reads were realigned onto the contig sequences to calculate the amount of shared paired-end relationships between each pair of contigs, and to weigh the rate of consistent and conflicting paired-ends, and to further construct the scaffolds. In order to find the most appropriate K-mer size for each sample, we first tested a 23-mer. If the resulting scaffold N50 was less than 100 kb under this setting, we further ran different K-mers (21-, 25-, 27, 29-, 31-, 33-, 35-, 37-, and 39-mer) and chose the K-mer that produced the largest scaffold N50 length. Gap filling was done with Gapcloser v1.12[11] and the paired-end information. Specifically, we searched for read pairs of which end was mapped to a unique contig and the other was located in the gap region. Thus, the gaps could be closed by a local assembly for these collected reads.

Assembly strategy using Allpaths-LG

All raw reads were introduced into Allpaths-LG, followed by correction of sequencing errors within reads, closure of short-fragment read pairs (inward), formation of an initial *de Bruijn* graph from these filled fragments, and disambiguation of the graph using paired-ends from the mate pair libraries as jumping libraries (outward). Assembly was run with default parameters and HAPLOIDIFY=TRUE.

Assembly strategies for external (OUT) genomes

Genomes from external sources were assembled by a variety of algorithms, Allpaths-LG[10], SuperNova[12], SOAPdenovo[9], MaSuRCA[13], Platanus[14], Meraculous[15], Spades[16], or Abyss[17] and gap-closed with PBJelly[18] and HiRise[19]. The individual assembly strategies for the newly released OUT genomes are reported in Supplementary Table 2.

<u>Genome assembly quality assessment</u>

Assembly quality for the 272 newly sequenced B10K genomes was assessed by contig N50, scaffold N50 and total assembly length.

<u>Genome completeness</u>

Genome completeness was measured with BUSCO (v3)[20] using aves_odb9 as the reference gene set for 285 species (236 B10K species and 49 OUT species). We measured three standard categories of BUSCO results, and obtained the following metrics.

1) Genome completeness based on Complete and single-copy BUSCOs (S).
2) Genome completeness based on Complete and duplicated BUSCOs (D).
3) Genome completeness based on Fragmented BUSCOs (F).

We then combined complete (S and D) and fragmentary (F) hits against BUSCO genes to assess the completeness degree of the genome.

DNA barcoding for quality control and species confirmation

Special caution was given to assure high quality data. We employed quality checks to first detect possible mislabeling due to lab errors or contamination during the sequencing process, and secondly to confirm the species identity of the sequenced samples. We amplified DNA barcodes from the same individual that was sequenced for its genome. Four PCR primer pairs were designed to amplify four candidate DNA barcodes of genes that had non-conserved regions across birds, the mitochondrial cytochrome c oxidase I gene (COI), the mitochondrial 16S ribosomal RNA gene (16S rRNA), the nuclear BDNF gene and the nuclear FAM222B gene (Supplementary Table 4). We also used their mitochondrial genomes to extract some other commonly used barcode genes (ND2, ND3, and CYTB). PCR-based analyses were performed on the 259 B10K genomes that passed assembly quality filters. The first test was to compare the barcode sequence to the corresponding mitochondrial or nuclear genomes to identify errors during library preparation or cross-contamination during the sequencing process. The barcodes confirmed all species matched their corresponding genomes.

Secondly, we took several steps to confirm the species identity. To confirm that the sequenced specimen belongs to the target species determined by the museum experts or collectors, we BLAST (v2.2.26) searched using our barcode against the COI sequences from the Barcode of Life Data (BOLD)[21] system and NCBI. The databases had COI barcodes for 184 of our target species.

1. If the COI sequence of the sample was >98% identical over >500 bp of the COI sequence of the target species, we considered the sample to belong to the target species. This confirmed 174 species.
2. If the COI of the best-matched species was a different species than the target species in the database, we considered the sequenced sample to be mislabeled. We found 4 samples with problems, which were subsequently tracked down to label switches during lab work. Using the BLAST results, we were able to correct these label switches.
3. If the COI sequence of the sample failed to match against the COI sequence of the target species and the best-matched species could not be determined (no high identity hit), we considered the sample to be contaminated. We discarded 6 genomes with this problem.

For the remaining 75 species without COI sequences in the databases, we used the other PCR barcodes (16S rDNA, BDNF, and FAM222B) or other mitochondrial regions from their mitochondrial genome assemblies (ND2, ND3, and CYTB) to confirm their identities. 45 species could be confirmed in this way.

The remaining 30 sequenced species did not have any published barcode information. Thus, we checked their K-mer distribution curves and the PCR chromatograms to evaluate the possibility of contamination. We found that 3 samples were problematic since their chromatograms had nested multicolor peaks indicating the possibility of contamination. The remaining chromatograms were clean.

In order to further evaluate potential contamination of the last 27 samples, we collected the toe-pads of the same species but from a different individual. DNA was extracted from historical museum specimens at the Centre for GeoGenetics at the University of Copenhagen, by digesting a subsample of toepad in a proteinase K containing buffer[22]. The digest was subsequently mixed 1:10 with a binding buffer[23] and centrifuged through Monarch DNA Cleanup Columns (5 µg) (New England Biolabs Inc. Beverly, MA, USA). DNA bound to the column was washed with 800 µl buffer PE (Qiagen, Hilden, Germany), then eluted using two washes in 20 µl buffer EB (Qiagen), each with an incubation of 5 min at 37 °C. These libraries were sequenced on BGI500 platform in PE100 or SE100. The barcode PCR products of the 27 samples were then compared against the nucleotide information of these toe-pads. According to the BLAST results, 4 samples were contaminated and 1 sample was mislabeled.

In summary, 246 sequenced species passed quality control after contamination and mislabeling tests (Supplementary Table 5 and 6). Among them, 10 species were already available on NCBI or provided by external labs and had better assembly quality statistics than the corresponding B10K genome. Thus, we replaced them and finally included 236 newly sequenced species into Phase II (Extended Data Fig. 1c).

Mitochondrial genome assembly and annotation

We conducted *de novo* assembly of the mitochondrial genomes (mtDNA) for the 363 samples using the mitochondrial genome assembler NOVOPlasty (v2.7.2)[24], which adopts a seed-and-extend algorithm to assemble mitochondrial genomes from whole genome sequencing data. The main steps were as follows (summarised in Extended Data Fig. 1d):

1. Preparation of raw sequencing reads for all 359 species (raw reads were not available for four publicly available samples (NCBI-003, NCBI-004, NCBI-011, NCBI-013)). The paired-end (PE) short read library with the largest number of sequencing reads for 359 samples (42 APP, 236 B10K, 49 OUT, 32 NCBI) was chosen for each sample.

2. Selection of starting seed sequence. The seed sequence used to start the assembly process was derived from one of the following sources: 1) ~650 bp COI barcoding sequences, which were generated by PCR amplification for quality control of some samples (please refer to "DNA barcoding for quality control and species confirmation"); 2) the complete mtDNA of Red Junglefowl (*Gallus gallus*, NC_040902.1); and 3) the longest mtDNA gene fragments available for the target species on NCBI;
3. *De novo* assembly. Mitochondrial genomes were assembled from small insert size libraries using NOVOPlasty with default parameters, that is with a K-mer=39 in most cases. If assembly failed for this setting, smaller K-mer sizes (23 or 19) and bigger memory allocations were also tested. Repeated trials with different combinations of sequencing libraries or seed sequences for non-circularised samples were carried out to achieve the best assemblies.
4. Selection of best mtDNA assembly. If multiple assemblies existed for one sample, the circularised mtDNA sequence with fewer ambiguous bases was regarded as the best quality assembly. If mitochondrial contigs were not circularised, the longest contig was selected.

MitoZ (v2.3)[25] was used to annotate the qualified mitochondrial genome using the vertebrate mitochondrial translation table. Only the longest contig for each sample was used for annotation, with the exception of B10K IDs B10K-DU-001-21 (Rufous Motmot, *Baryphthengus martii*) and B10K-DU-001-62 (Paradise Jacamar, *Galbula dea*), where the first contig was identified as an assembly artifact. Contigs were reoriented to the first position of trnF, if it was included in the assembled contig, by using the "sort" option of MitoZ. NAD3 is known to contain a frameshift insertion at position 174 in some birds[26]. Therefore, NAD3 genes were extracted and aligned using MAFFT (v7.4)[27] to identify sequences with the NAD3-174 frameshift +1 insertions and record them in the GenBank submissions. Nonsense mutations in protein-coding genes were masked from the submitted GenBank records but the original codon information is given as a note on the corresponding sequence. Contigs with no gene present were filtered.

<u>Protein coding genes annotation</u>

Annotation of protein coding genes was conducted with a homology-based method. Given the importance of the quality of the reference gene set in the homology-based gene prediction, we first carefully generated a reference gene set, which was then used to annotate protein coding regions of the 363 avian genomes. The reference gene set consisted of 20,194 avian genes, of which 12,292 genes were orthologous between chicken

and zebra finch, 3,025 genes were unique to chicken and 4,877 genes were unique to zebra finch (Supplementary Table 7). This primary reference gene set was complemented with 20,169 human genes, and 5,257 genes derived from 25 avian species with published transcriptomes. We applied this primary gene set to all 363 qualified genomes and then supplemented these annotations with non-redundant annotations from the supplementary human gene set and the transcriptomes set.

A. Primary reference gene set.

Generation of primary reference gene set. In order to identify orthologs between chicken and zebra finch reference genomes, the Ensembl gene sets (release 85) of chicken (*Gallus gallus*, GCA_000002315.2) and zebra finch (*Taeniopygia guttata*, GCA_000151805.2) were mapped against the chicken-zebra finch whole-genome alignment from the UCSC Genome Browser using a custom Blastp (v2.2.26) pipeline with default parameters. A total of 12,350 orthologous gene pairs between chicken and zebra finch were identified based on synteny. Since only one gene in each orthologous gene pair from either chicken or zebra finch should be included into the reference gene set to reduce redundancy, the protein sequences of the orthologous genes in chicken and zebra finch were BLAST-aligned to the human protein sequences (hg38). The aligned lengths of the chicken or zebra finch genes relative to the human genes were recorded. For a given orthologous gene pair, the gene with the higher proportional alignment to the human protein sequence was added to the reference gene set for downstream annotation. In cases where the aligned rates were equal or where no human homolog existed, the zebra finch ortholog was chosen since most of the 363 avian species were Neoavian.

This procedure included 5,451 chicken and 6,899 zebra finch genes of the 12,350 orthologous gene pairs in the reference gene set. Protein coding genes in chicken and zebra finch that did not contribute to the 12,350 orthologous gene pairs were also added to the reference gene set, 3,158 genes unique to chicken and 5,084 genes unique to zebra finch. The primary reference gene set was then filtered by removing genes with length <150bp, genes harboring transposons or retrotransposons, and genes with a single exon but without any evidence of function listed on either the InterPro (version 5.24-63.0)[28], SwissProt (release-2018_07)[29] or KEGG (release 81)[30] databases. Finally, this primary reference gene set contained 20,194 avian genes, of which 12,292 genes were orthologous between zebra finch (6,842 genes) and chicken (5,450 genes), 4,877 genes were unique to zebra finch and 3,025 genes were unique to chicken (Supplementary Table 7).

Annotation with the primary reference gene set. The primary reference gene set was used for the homology-based gene prediction for all 363 avian genomes through the following four main steps:

1. Rough alignment: The protein sequences of the reference gene set were aligned to each genome by tblastn (v2.2.2)[31] with an E-value cut-off of $1e^{-5}$, and the result hits were linked into candidate gene loci with genBlastA (v1.0.4)[32]. The candidate loci with homologous block length <30% of length of query protein were removed.
2. Precise alignment: The genomic sequences of candidate gene loci were extracted, including the intronic regions and 2000 bp upstream/downstream sequences. We used GeneWise (wise2.4.1)[33] to predict more precise gene models in these regions. After translating the predicted coding regions into protein sequences, we ran MUSCLE (version 3.8.31)[34] for each pair of predicted protein and reference protein. The predicted proteins with length of <30 amino acids or percent identity of <40% were removed, as well as the pseudogenes (genes containing >2 frame shifts or premature stop codons) and retrogenes.
3. Building a non-redundant gene set: The output of GeneWise could include redundant gene models overlapping at the same genome regions. Hierarchical clustering[35] was applied to build a non-redundant gene set. Gene models that overlapped in >40% of their coding sequence were clustered into one group and the one with the highest identity to the reference proteins were retained.
4. Removing the highly duplicated genes: Through the above steps, we obtained 19,824 genes for zebra finch and 19,612 genes for chicken, an increase relative to their Ensembl gene sets (17,421 for zebra finch, and 15,495 for chicken). By comparison, we found that the main source of these differences came from several highly duplicated gene family expansions. These highly duplicated genes were mostly single exon genes that overlapped with repeat elements. Therefore, we removed the annotated genes in all 363 birds if they had >10 duplications, were single exon genes, and contained >70% repeat sequences in the coding region.

B. Supplemental human gene set.

Generation of the human gene set. Given the high quality of human gene annotations from Ensembl (GRCh38.85), the complete gene set (20,421 genes, hg38) was added to the reference gene set. A total of 20,169 genes remained after filtering out genes with length <150bp, genes harboring transposons or retrotransposons, and single-exon genes without any evidence of function in either the InterPro (version 5.24-63.0), SwissProt (release-2018_07), or KEGG (release 81) databases.

Annotation with the human gene set. This supplemental human gene set was used for homology-based gene prediction for all 363 avian genomes following the same four steps outlined for the primary reference gene set. As expected, most annotated genes based on the supplemental human gene set overlapped with the results of the primary reference gene set. In these cases, we only kept the newly annotated loci from this supplemental set that did not overlap with the primary one.

C. *Supplemental transcriptome gene set.*

Generation of the transcriptome gene set. Published avian RNA-seq data of 71 samples were collected from NCBI (Supplementary Table 8). We only selected the RNA-seq data obtained from non-pathological samples from each NCBI project containing only a single species. The 71 transcriptomes came from 25 species in 16 families and 7 orders. We conducted reference-based transcriptome assemblies for 7 species that had whole-genome sequencing data available from Phase II species using TopHat (v2.1.1)[36] and Cufflinks (v2.2.1)[37], while the others were *de novo* assembled using Newbler (v2.9)[38] for 454 sequences and Trinity (version trinityrnaseq_r20140717)[39] for Illumina sequences.

We built a credible transcriptome gene set by filtering out redundant and low-quality results as follows. First, candidate transcripts from the *de novo* assembly were removed if they overlapped with the one supported by reference-based assembly, the primary reference gene set, or supplemental human gene set. Second, candidate transcripts from the *de novo* assembly with ORF length <150 bp were removed. The remaining transcripts were clustered using cd-hit (v4.6.6)[40] to further remove redundancy and candidate transcripts harboring transposons or retrotransposons, or lacking a function listed on the database of InterPro (v5.24-63.0) were removed. Finally, the supplemental transcriptome gene set contained 5,257 transcripts.

Annotation with the transcriptome dataset. This supplemental transcriptome gene set was used for homology-based gene prediction for all 363 avian genomes following the same four steps outlined for the primary reference gene set. As expected, most annotated genes identified using this reference gene set overlapped with the results of the primary reference gene set and the supplementary human gene set. In these cases, we only kept the new annotated loci from this supplementary set without any overlap with the previous annotation results.

<u>Repeat annotation</u>

Tandem repeats and transposable elements (TEs) were annotated across all 363 avian genomes. We used Tandem Repeats Finder v4.07b[41] to identify tandem repeats, and used both the homology-based and the *de novo* approaches to identify TEs. The homology-based repeat

annotation of all 363 species were done by RepeatMasker (open-4.0.7)[42] (http://www.repeatmasker.org, with parameters "-nolow -no_is -norna -engine ncbi -parallel 1") at the DNA level based on the Repbase library (v20170127). The de novo repeat annotation of all 363 species were done by RepeatModeler (open-1-0-8)[43] (http://www.repeatmasker.org) with default parameters to first build a de novo repeat library for each assembly. Further, we used the de novo repeat library with RepeatMasker (open-4.0.7) to predict repeats for each species. All the above results were merged into a unified set for each bird (Supplementary Table 1). We calculated mean TE content across orders with more than one sequenced representative and standard deviation to identify orders with variable TE content. We reconstructed the ancestral state of total TEs with maximum likelihood using the fastAnc function in the R package phytools (v0.7-20)[44].

Cactus whole-genome alignment

We generated a phylogenetic hypothesis to use as a guide tree for Cactus by extracting ultraconserved element (UCE) regions[45] from each of the 363 bird assemblies following a standard protocol[46] (https://phyluce.readthedocs.io/en/latest/tutorial-three.html). Specifically, we identified UCE regions using PHYLUCE (at commit 69e7849), sliced regions ± 500 bp sequence flanking each UCE locus, aligned slices with mafft (v7.313)[27], and trimmed the resulting alignments with TrimAl (v1.4.rev15)[47]. We then created a data matrix containing only those alignments with >75% of the 363 bird species, and we concatenated all alignments within this data matrix. We generated a temporary tree to check for obviously incorrect tip placements using PAUP (v4a164)[48]. After observing no obvious errors in the temporary tree, we performed maximum likelihood (ML) inference on the concatenated dataset using ExaML (v3.0.9)[49] on an HPC system assuming a general time reversible model of rate substitution, gamma-distributed rates among sites, and five tree searches.

We ran Cactus (at commit f88f23d) on the Amazon Web Services (AWS) cloud, using the AWSJobStore of Toil to store intermediate files. We used an auto-scaling cluster which varied in size during the course of the alignment, but used a combination of c3.8xlarge (high-CPU) and r3.8xlarge (high-memory) worker nodes. A MAF format file was derived from this alignment using a parallelised version of the command `hal2maf --onlyOrthologs --refGenome Gallus_gallus`.

Chicken and zebra finch were marked as preferred outgroups, meaning that they would be chosen as outgroups if they were candidates, to ensure that a high-quality assembly was almost always available as an outgroup. Three genomes were used as outgroups to the avian tree: Common Alligator (*Alligator mississippiensis*, v. ASM28112v4), Green Anole (*Anolis carolinensis*, v. AnoCar2.0), and Green Sea Turtle (*Chelonia mydas*, v. CheMyd1.0). These outgroups were not included in the alignment, but used only to provide

outgroup information for subproblems near the root (by using the `--root` option to select only the avian subtree).

Intron dataset construction

Introns of the 15,671 orthologs among 363 species with conserved synteny with chicken as the reference generated from our new pipeline were extracted from the Cactus alignment using the following steps (Extended Data Fig. 5b):

Step 1: Masking potential coding regions within the introns of chicken. We downloaded RNA-seq data of chicken from NCBI (three runs: SRR7523562, SRR5457066, and SRR4292804). By mapping RNA-seq reads onto Gallus_gallus-4.0 (GCA_000002315.2), we considered the regions of the intron that were covered by RNA-seq reads with mapping depth ≥3 as potential coding regions and masked them. If the length of the remaining intron fragments was <300 bp, we filtered out these fragments.

Step 2: Pre-extraction of orthologous introns from alignment based on the gene models of chicken. To obtain introns with conserved boundaries between chicken and other birds, we prepared a BED file including the coordinates of the 5' and 3' ends of all qualified intron fragments according to the gene models of chicken. With this BED file, we extracted the corresponding coordinates for the other birds based on the Cactus alignment. We only used the 1:1 aligned intron fragments for the next step. For each intron fragment in every species, we considered the intron fragment missing if it matched any of the following conditions:

1. One of the flanking sequences could not be located in the species' Cactus alignment.
2. According to the annotation results of this species, the corresponding coordinates of the flanking sequences did not belong to the correct pairwise orthologous gene.
3. According to the annotation results of this species, the corresponding coordinates of the flanking sequences did not belong to the same intron fragment.

Step 3: Final extraction of alignments for orthologous introns and masking all non-intron regions in any species. For each intron fragment, we extracted the alignments of the conserved introns from the Cactus alignment based on the qualified chicken's coordinates in Step 2. Given that the aligned regions of the other 362 birds could be located in exons or across the exon-intron-boundaries, the extracted alignments may contain some non-intron

regions. Thus, we masked these non-intron regions of 362 birds as gaps according to their respective gene models.

Step 4: Removing repeats and gapped regions in the intron blocks. We then used the annotated repeat elements of chicken to remove any repeat regions and filtered out gapped loci (>99% missing) from the extracted alignments.

Codon preference

To examine the variation in codon usage across birds, we calculated the relative synonymous codon usage (RSCU) for 59 codons (excluding the single codons Met, Trp, and the three stop codons) of the protein-coding genes of all 363 bird species (Extended Data Fig. 4d). The RSCU is the ratio of the observed frequency of a codon to the expected frequency of a codon if all the synonymous codons for a particular amino acid were used equally[50]. Under this definition, if the RSCU value of a codon is greater than one, the codon is more frequently used than expected, whereas if the RSCU value of a codon is less than one then the codon is less frequently used than expected. To summarise the overall variation in codon usage between species, we conducted a correspondence analysis on RSCU values[51] across all 363 species.

To assess the differences between the Passeriformes and other species at the gene level, we compared the mean values of the effective number of codons (Nc)[52] for each ortholog. Nc quantifies the departure of a gene from the random usage of synonymous codons and is related to the amount of entropy in the codon usage of a sequence. It reaches the maximal value of 61 when all codons are used equally and its minimal value of 20 when only one codon is used per amino acid[52]. Nc was calculated for each gene with CodonW (v1.4.2, J Peden, http://codonw.sourceforge.net/)[53].

Analyses of gene duplication, gene loss and pseudogenes

The increased taxon sampling along the bird tree of life allowed to more comprehensively study genetic and functional diversity of previously reported genes from Zhang et al. 2014[3,54] and Yuri et al. 2008[54].

Detecting gene loss in avian genomes

Zhang et al., 2014 identified 640 human genes that were present in non-avian reptiles but lost in the modern birds. Using all 363 avian genomes, we checked the presence/absence of these genes with the same method as Zhang et al., 2014. A gene was considered as present when their coding frame could be annotated without frame-shifts or premature stop codons.

Rhodopsin/opsins and vision

Zhang et al., 2014 compared various genes associated with phenotypes and physiological pathways between birds and mammals. We re-examined genes related to the vertebrate visual opsins in 48 birds: rhodopsin (*RH1*) and conopsins (*RH2*, *OPN1sw1*, *OPN1sw2*, and *OPN1lw*). We downloaded the protein sequences of five visual opsin genes from GenBank (NP_001025777.1, NP_990821.1, NP_990769.1, NP_990848.1 and NP_990771.1 of chicken; NP_001070163.1, NP_001070164.1, NP_001070172.1, NP_001070165.1 and NP_001070170.1 of Zebra finch) and used tblastn to search potential opsin sequences in all 363 birds. GeneWise was performed on these potential sequences to predict gene structures. After translating predicted genes into protein sequences, we used MUSCLE to align the predicted proteins to their reference protein. The predicted proteins with length of ≥30 amino acids and percent identity of ≥40% were accepted. We further checked whether the predicted opsin genes overlapped with any genes in the full protein coding genes annotation result for each species. We only kept the annotated opsin gene if it had ≤40% overlap with the genes having different functions in the full protein coding genes annotation result.

We divided the annotated genes into five categories: 1) Functional gene; 2) Sequence with premature stop codons; 3) Sequence with frameshift; 4) Partial sequence; and 5) Gene not found. Functional genes were annotated opsin genes without any frameshifts or premature stop codons. Otherwise, if a sequence contained premature stop codons or frameshifts, making them likely dysfunctional (pseudogenes), we annotated them as separate categories. These frameshifts and/or partial sequences could be due to sequence errors or an incomplete assembly, respectively, rather than gene loss. Therefore, we relaxed the acceptable thresholds mentioned above in the gene annotation pipeline for species without any annotated genes into: length of ≥10 amino acids and percent identity of ≥70%. Candidate genes with these characteristics were regarded as the partial sequences. Genes were considered as not found, if none of these criteria were met.

Growth hormone (GH) duplication in Passeriformes

Yuri et al., 2007[54] uncovered the duplication of the growth hormone gene (*GH*) into copies *GH_L* and *GH_S* in 24 Passeriformes birds. We investigated the distribution of *GH* copies among all 363 genomes, including 173 Passeriformes. We built a maximum likelihood gene tree from the *GH* sequences after selecting the most appropriate model of sequence evolution and with 1000 ultrafast bootstraps in IQ-TREE (v1.6)[55–57].

Loss of Cornulin (CRNN) in songbirds

Cornulin (*CRNN*) has evolved in a common ancestor of terrestrial vertebrates[58,59]. In humans and chicken *CRNN* is expressed in the stratified epithelium of the esophagus and, at lower levels, in keratinocytes of the oral epithelium, the epidermis of the skin and skin appendages[58,60]. The protein encoded by *CRNN* undergoes crosslinking by transglutamination to increase the mechanical resilience of the outer layers of these stratified epithelia[58]. *CRNN* of birds is located between the genes *EDDM* and *EDNC* on its 5'-side and trichohyalin-like/scaffoldin and *S100A11* on its 3'-side. We examined the presence or absence of *CRNN* in the genomes of 363 birds and specifically investigated the locus flanked by *EDDM* and *S100A11*, both of which show high sequence conservation in birds.

Supplementary Results

Genome assembly quality

The contig N50 of most species ranged from 2 to 100 kb (mean 37 kb). The scaffold N50 of most species ranged from 50 kb to 5 Mb (mean 701 kb). (Supplementary Table 1, Interactive Supplementary Figure 1 https://genome-b10k.herokuapp.com/main). The total assembly length of most species was around 1 Gb, as for most birds[61] (mean 1.08 Gb). The 13 genomes discarded due to poor quality (Supplementary Table 3) had the following metrics:

1. 9 genomes with scaffold N50 <10 kb;
2. 4 genomes with total length <0.9 Gb.

For the published NCBI and APP genomes, contig N50 ranged from 7 to 439 kb (mean 58 kb), scaffold N50 ranged from 30 kb to 82 Mb (mean 10 Mb) and total length was on average 1.14 Gb. OUT genome contig N50 ranged from 7 to 225 kb (mean 72 kb), scaffold N50 from 35 kb to 22 Mb (mean 6.3 Mb) and total assembly length was on average 1.10 Gb.

Genome completeness

Genome completeness was measured for 285 species (236 B10K species and 49 OUT species). Genomic compleness based on complete and single-copy BUSCOs (S) was 88.0%

on average, and ranged from 42.8% (Puerto Rican Tody, *Todus mexicanus*) to 94.4% (False Whistler, *Rhagologus leucostigma*). Genome completeness based on Complete and duplicated BUSCOs (D) for 285 species was 0.9% on average and ranged from 0.2% (Common Sunbird Asity, *Neodrepanis coruscans*) to 2.4% (Bearded Manakin, *Manacus manacus*). Genome completeness based on Fragmented BUSCOs (F) for 285 species was 5.7% on average and ranged from 2.7% (Superb Lyrebird, *Menura novaehollandiae*) to 20.8% (Sunda Bush Warbler, *Horornis vulcanius*).

Here, we combined complete (S and D) and fragmentary (F) hits against BUSCO genes to assess the completeness of the genome. Only 18 species had <85% completeness and 6 species had <70% of BUSCO genes. More detailed information can be found in Supplementary Table 1 and Interactive Supplementary Fig. 1 https://genome-b10k.herokuapp.com/main.

Mitochondrial genomes

Of 359 samples (raw reads were not available for four publicly available samples), 13 samples (3.62%) did not produce any mtDNA contigs irrespective of the assembly settings. For 216/359 (60.17%) samples, multiple assemblies existed, of which the one with fewer ambiguous bases was chosen. 14 out of those 216 contigs were circularised but had two contigs, which were manually merged into a single contig based on overlapping bases. For 130/359 (36.21%) samples, the mitochondrial assembly did not circularise and the longest contig was selected. A total of 228 mitochondrial genomes were annotated with the complete set of 37 genes, the remaining having a subset of those genes (Supplementary Table 1).

Repeat content

When averaged across orders with more than one sequenced representative, 96% of species had a TE content (% of bp per genome) lower than 15%, with Piciformes species containing more TEs than other birds (Welch Two Sample t-test, p-value = $9.983e^{-05}$, Extended Data Fig. 2a). After examining each TE category, we found that the differences between Piciformes and other orders were mainly due to the content in LINEs (Welch Two Sample t-test, p-value = $3.595e^{-05}$, Extended Data Fig. 2b). We measured differences between species within the same order and found that Bucerotiformes showed the highest value (Standard deviation value of the total TEs and LINEs is 7.33 and 7.29, respectively), which was caused by species-specific expansion of LINEs in two species, Common Scimitarbill (*Rhinopomastus cyanomelas*) and Common Hoopoe (*Upupa epops*) (Extended Data Fig. 2c,d). Ancestral state reconstruction of TE content further confirmed the expansion of TEs in the common ancestor of the Piciformes, as well as the two Bucerotiformes species (Extended Data Fig. 2e).

Cactus whole-genome alignment

Cactus aligned 981 Mb (93.7%) of the chicken genome and 1.17 Gb (94.8%) of the zebra finch genome to at least one other species. The proportion was much greater for functional sequence: e.g. for chicken genes identified by BUSCO, 97.5% had an alignment to turkey (also a galliform bird like chicken) covering the majority of their bases, and 92.5% of bases of chicken genes had an alignment to ostrich (a palaeognathae).

Ortholog identification

The orthology identification pipeline identified 22,833 homologous groups, which include all possible gene pairs within and between species. These homologous groups can be used to study the evolution of particular genes including all duplications. Step 3 of the pipeline resulted in 15,671 orthologs, including one-to-one orthologs, and ancestral and novel copies from one-to-many or many-to-many orthologs, where those copies could be distinguished.

Effect of adding species on orthologs with conserved synteny with chicken. When only including 48 birds, we obtained 15,232 orthologs between chicken and other birds, compared to the 15,671 orthologs when scaling up to 363 species, which translates to a 3% increase (439 additional orthologs). The reason that some of these additional orthologs were missing from the previous 48 birds was because many of them were lineage-specific orthologs that are not present in the 48 birds. For example, from 48 to 363 birds, sampling in Galliformes increased from 2 (chicken and turkey) to 11 species. This increased the detection of orthologs that were not limited to chicken and turkey. 224 of 439 orthologs were lineage-specific orthologs between chicken and some Galliformes other than turkey. In the 48 bird dataset, these 224 orthologs were missed.

Dataset sizes for different genomic categories

In order to quantify the dataset sizes of orthologous regions of different functional categories (general length of the whole-genome alignment, coding sequences, introns), we compared the new dataset against the corresponding datasets from the 48 birds analyzed in Phase I of the project.

Whole-genome alignment. The Cactus alignment produced 981 Mb of aligned sequence across the whole genome. In Phase I, the whole-genome alignment based on MULTIZ was 393.7 Mb long. This corresponds to a 149% increase. When requiring 90% of all species to be aligned (>326 of the 363 species being aligned), Cactus produced 546 Mb of alignment.

The MULTIZ alignment of Phase I produced 322.15 Mb with 90% of species being aligned (no more than 5 missing species out of 48). This corresponds to a 69% increase.

Coding sequences. According to the orthologs with conserved synteny with chicken, we summed the length of the (unaligned) coding region of 15,671 chicken orthologous genes to a total of 23.79 Mb. This compares to the exons extracted during Phase I of 8,295 orthologous genes, which were produced using the RBH-based pipeline. For comparison, we summed the unaligned length of the chicken sequence in the 8,295 orthologs before any filtering, 12.9 Mb. This corresponds to an 84.4% increase of unaligned coding sequence.

Intron sequences. A total of 140.70 Mb of intron sequence were extracted using the procedure described in the section Intron dataset construction. In Phase I, introns were identified in between a small set of 2,516 orthologous coding sequences, with a total length of 19.26 Mb aligned intronic sites[4]. This corresponds to a 631% increase in aligned intron length.

GC content

A Welch Two Sample t-test was performed between Passeriformes and non-Passeriformes using the summed GC content for each species. A principal component analysis (PCA) performed on a matrix consisting of GC content in the coding regions of the orthologs with conserved synteny with chicken (Supplementary Table 12) showed that the 164 included species of Passeriformes (out of 176 species, excluding 9 species with more than 40% missing data) clustered separately from the remaining birds (Extended Data Fig. 4a). Further, when studying 14,229 of 15,671 orthologs that were present in at least 20 birds of both Passeriformes and non-Passeriformes, we found that 10,246 of 13,700 orthologs (74.79%) showed a higher average GC content in Passeriformes, of which 8,434 orthologs (82.32%) had significant p-values. In the remaining 3,454 orthologs (25.21%) with a lower average GC contents in Passeriformes, 2,120 of 3,454 orthologs (61.38%) had significant p-values. This indicates that Passeriformes and non-Passeriformes generally differ in their GC content, although genes were not consistently higher or lower in one of the groups.

Codon preference

We found 32 codons with RSCU value greater than 1 (the codon is more frequently used than expected), of which 21 were codons ending in G or C (Extended Data Fig. 4d). The remaining 27 codons had a RSCU value less than 1 (the codon is less frequently used than expected), of which 19 were codons ending in A/T. We defined codons with RSCU value greater than 1.6 as over-represented codons and those with RSCU value less than 0.6 as

under-represented codons. In total, there were two over-represented codons (CTG and GTG) and eleven under-represented codons (ATA, ACG, TTA, TCG, CAA, CTA, CCG, CGA, CGT, GTA, and GCG) (Extended Data Fig. 4d).

The plot of the correspondence analysis of RSCU values shows the distance between species in RSCU values on two axes (Extended Data Fig. 4b). The first dimension reflected the primary factor that explained 78.18% and the second axis explained 14.82%. Passeriformes and other species separated along this first axis. The corresponding distribution of synonymous codons showed the separation of C or G-ending codons and A or T-ending codons along the first axis (Extended Data Fig. 4c). This indicates that the variation in synonymous codon usage among species was based on their nucleotide content (e.g. GC content). The correlation between the GC content of the third codon position (GC3) of each bird and their location on the primary axis of the correspondence analysis was highly significant (Pearson's correlation, R^2=0.9, p-value=4.1e^{-184}, Extended Data Fig. 4e), indicating that the variation in codon usage is strongly correlated with the GC3 content (the usage of G or C-ending codons).

We found that the mean effective number of codons (Nc) in Passeriformes was significantly smaller than that of other birds (Paired Sample T-Test, p-value < 2.2e^{-16}), suggesting that Passeriformes use less codons than expected from the random usage of synonymous codons (Extended Data Fig. 4f). Therefore, the codon bias in the Passeriformes is stronger than that in non-Passeriformes.

<u>Analyses of gene duplication, gene loss and pseudogenes</u>

Detecting gene loss in avian genomes

A gene was considered as present when their coding frame could be annotated without frame-shifts or premature stop codons. With these criteria, we confirmed the absence of these 640 genes in the 48 bird genomes reported in Zhang et al., 2014[3,54], but we found that 142 of these 640 genes were present in at least one other species of the remaining 315 species (Supplementary Table 9). This result indicates that the initial detection of absence was correct using this method but that the denser sampling can provide increased insight into true Aves-wide losses of genes or that improved assembly quality has identified additional genes.

Rhodopsin/opsins and vision

Rhodopsin *RH1* and *RH2* sequences were present in all bird species, but were incomplete or pseudogenised in a few species (Supplementary Table 10, Extended Data Fig. 3). The three conopsin genes were more variable. *OPN1sw2* and *OPN1lw* were functional sequences only in a few species and were completely lost in many species. *OPN1sw1* was

functional in more than half of 363 birds, especially in Passeriformes. We also found frameshifts within this gene in 12 species that were distributed across the phylogeny, including the previously reported frameshifts of the Yellowhead (*Mohoua ochrocephala*)[62].

Growth hormone (GH) duplication in Passeriformes

Using the gene annotation of 363 avian genomes, we found that the *GH* gene was present as two copies in most of the 173 Passeriformes, except for 12 species, which had only one copy of *GH* (7 of 12 Passeriformes only had *GH_L* and the remaining 5 had only *GH_S*). The absence of two copies in 12 Passeriformes was likely due to incomplete assembly, rather than a true loss, because we found that the regions around the missing copies had had poor assembly quality, i.e., the surrounding genes were on a short scaffold or at the end of the scaffold. The maximum likelihood gene tree for copies of *GH* is consistent with the Yuri et al., 2007[54] study in that both *GH_L* and *GH_S* of Passeriformes formed separate clades (Extended Data Fig. 6, tree file Extended_Data_Figure_6.newick.tre is available under doi:10.17632/fnpwzj37gw). This result provides further evidence for the ancestral duplication of the *GH* gene in the common ancestor of Passeriformes.

Loss of Cornulin (CRNN) in songbirds

CRNN was found to be inactivated by mutations or entirely absent from the *EDDM* to *S100A11* region in three clades of birds: Accipitriformes (eagles and related birds of prey), Phalacrocoracidae (cormorants) and Passeri (songbirds) (Extended Data Fig. 8a). Loss of *CRNN* in the esophageal epithelium may affect the primary function of the esophagus, i.e. to provide a path for the transport of food from the mouth to the stomach; however, comparative analyses of the interactions between the esophageal epithelium and ingested food in cornulin-deficient and cornulin-proficient species have not been reported to the best of our knowledge. By contrast, a secondary function of the esophagus was shown to be directly related to a characteristic trait of songbirds, i.e. the ability to produce pure-tone song. Studies in the Northern Cardinal (*Cardinalis cardinalis*) have demonstrated that songbirds utilise an acoustic filter consisting of the upper esophagus and the pharynx (oropharyngeal-esophageal cavity, OEC) to eliminate overtones (upper harmonics) from the tones that are produced in the syrinx[63]. Acting as a Helmholtz resonator, the volume of the OEC determines the frequency of the tones that are filtered. Accordingly, changes in OEC volume are necessary to produce pure tones of different frequencies. The OEC volume is primarily altered by increasing or decreasing the diameter of the upper esophagus, which depends on: 1) the flexibility of the esophagus and 2) fine-tuned movements of the hyoid apparatus. Cornulin contributes to epithelial cornification and thereby decreases the mechanical flexibility of the esophageal epithelium whereas absence of cornulin favors flexibility. Thus, the loss of *CRNN* after the divergence of the songbird lineages from the suboscine lineage

(Extended Data Fig. 8a) and the role of the esophagus in pure-tone song suggests the following evolutionary model: 1) The loss of *CRNN* in the last common ancestor of songbirds led to a decrease in rigidity of the esophageal epithelium; 2) This allowed fast, fine-tuned changes in the diameter of the upper esophagus and rapid changes in volume of the OEC; and 3) Subsequently, movement coordination between the OEC-expanding hyoid skeleton, the vocal organ and breathing evolved to facilitate an effective resonance filter which eliminates overtones, making possible pure-tone song over a range of fundamental frequencies (Extended Data Fig. 8b). Additionally, as-yet-unknown changes in gene expression have likely modified the esophagus in songbirds.

<u>Lineage-specific sequences based on whole-genome alignments</u>

The length of the lineage-specific elements and the size of the ancestral "genome" of the MRCA of the 37 bird orders are given in Supplementary Table 13.

Lineage-specific insertions and deletions identified with the Cactus alignment can be checked with assembly results and mapping of raw reads. To illustrate this process, we provide an example of a lineage-specific 36 bp insertion in Southern Cassowary (*Casuarius casuarius*) (Extended Data Fig. 7a). This insertion is located in Scaffold_56: 5,014,116-5,014,152 bp. This insertion is absent in the close relative Okarito Brown Kiwi (*Apteryx rowi*) in this region. Based on the coverage of raw sequencing reads mapping, we can detect reads spanning the 36 bp insertion in *Casuarius casuarius* that support the presence of this insertion, while all reads in *Apteryx rowi* do not support an insertion in the orthologous region.

We found that branch length (a proxy for divergence time) between the MRCA of a bird order and its parental node correlates with the amount of lineage-specific sequence (Extended Data Fig. 7b). An outlier is Tinamiformes, which have a low proportion of lineage-specific insertions relative to the long branch connecting the Tinamiformes MRCA with the parent node.

A total of 154 Passeriformes-specific genes were identified (Supplementary Table 14). According to the functional annotation, the three Passeriformes-specific genes that were present in the highest number of Passeriformes were 1) *DNAJC15* (DnaJ Heat Shock Protein Family Hsp40 Member C15) in 131 of 173 sequenced passerines (hereafter *DNAJC15-like*, see main text), 2) *COX5B* (nuclear-encoded mitochondrial gene) in 115 of these species, and 3) *SPAG7* (Sperm Associated Antigen) in 106 of 173 species.

We evaluated the synteny of the putative Passeriformes-specific gene DNAJC15-like (*DNAJC15L*) with seven flanking genes in all 363 birds (Extended Data Fig. 7c). The conserved synteny of the flanking genes without the presence of DNAJC15-like was

confirmed in all non-Passeriformes birds. In the main text (Fig. 2c), we show this synteny information in 8 Passeriformes [(New Caledonian Crow (*Corvus moneduloides*, Corvidae), Groundpecker (*Pseudopodoces humilis*, Paridae), Green Hylia (*Hylia prasina*, Scotocercidae), Eurasian Blackcap (*Sylvia atricapilla*, Sylviidae), Dark-eyed Junco (*Junco hyemalis*, Emberizidae), Many-colored Rush Tyrant (*Tachuris rubrigastra*, Tachurididae), Silver-breasted Broadbill (*Serilophus lunatus*, Eurylaimidae), Rifleman (*Acanthisitta chloris*, Acanthisittidae))] and 9 non-Passeriformes from 9 orders [Budgerigar (*Melopsittacus undulatus*, Psittaciformes), Golden Eagle (*Aquila chrysaetos*, Accipitriformes), Imperial Crested Ibis (*Nipponia nippon*, Pelecaniformes), Black-legged Kittiwake (*Rissa tridactyla*, Charadriiformes), Chimney Swift (*Chaetura pelagica*, Caprimulgiformes), Nicobar Pigeon (*Caloenas nicobarica*, Columbiformes), Muscovy Duck (*Cairina moschata*, Anseriformes), Red Junglefowl (*Gallus gallus*, Galliformes), and Elegant Crested Tinamou (*Eudromia elegans*, Tinamiformes)].

Selection analysis on whole-genome alignments

Realignment of conserved sites

The distribution of differences in score (of the realigned score relative to the original score) is shown in Extended Data Fig. 10c: 52% of scores were exactly identical, while 93% were within a range of 1.0 from the original score value (i.e. an order of magnitude in p-value). 8.4% of conserved sites had a realignment score that dropped below the significance threshold after realignment; however, most of these cases were only slightly above the threshold to begin with (median original score of 2.26, mean 2.41).

Comparison to a 48-way alignment

We compared the distribution of phyloP conservation scores between the 363-way alignment to the 48-way Cactus alignment and the 53-way MULTIZ alignment to investigate power to detect conserved sites. The 48-way had slightly less power than the larger alignments, as expected given the fewer species involved (a comparison of the distribution of scores is available in Extended Data Fig. 10d).

Supplementary Tables

All Supplementary Tables are available on Mendeley Data (doi:10.17632/fnpwzj37gw).

Supplementary Table 1. Sample information, basic statistics for the assemblies, of protein-coding gene annotations, and of mitochondrial genome assembly and annotation for all 363 genomes of Phase II of the B10K. The statistics can also be visualised interactively with https://genome-b10k.herokuapp.com/main. The spreadsheet has the following sections:

1) Taxonomy: B10K ID, Order, Family, Species latin name, Species common name, IUCN Red List Assessment
2) Sample information: sequenced tissue, BioProject accession number, etc.
3) Basic statistics for the assembly of each species: Contig N50 and L50, Scaffold N50 and L50, Total assembly length, Length and proportion of gaps, BUSCO results, Sequencing depth.
4) Basic statistics for the annotation of each species: Gene number, Mean gene length, Mean CDS length, Mean exon number and length, Mean intron number and length, Mean intergenic length, etc.
5) Mitochondrial genome assembly & annotation: completeness, Total assembled length, Largest contig length, Number of assembled contigs, Number of assembled contigs, Largest contig protein coding gene number, Largest contig tRNA gene number, Largest contig rRNA gene number, etc.

Supplementary Table 2. DNA extraction, sequencing strategy and assembly for strategy 49 OUT genomes and contact information for the individual genome contributors.

Supplementary Table 3. Basic assembly statistics of 13 genomes that were discarded due to poor quality.

Supplementary Table 4. Primer sets for species confirmation.

Supplementary Table 5. Basic statistics of 13 genomes with suspected contamination/mislabelling. *For the Northern Brown Kiwi (*Apteryx mantelli*), a published genome (code NCBI-005) was available to substitute this genome.

Supplementary Table 6. Summary of the species confirmation of newly sequenced species for Phase II.

Supplementary Table 7. Primary reference gene set for gene annotation.

Supplementary Table 8. Transcriptome samples used in building the transcriptomic gene set. N = no whole genomes available and transcripts were *do novo* assembled. Y = whole genome available for the species, which was the reference for transcript assembly. Y* are species for which a whole-genome assembly now exists but which were not available at the time of the generation of the supplementary transcriptome gene set. These transcriptomes were therefore *de novo* assembled.

Supplementary Table 9. Status of 142 genes that were previously defined as lost in the ancestor of modern birds but were now found in at least one of the new bird genomes.

Supplementary Table 10. Summary of the states of the five visual opsins (*RH1*, *RH2*, *OPN1sw1*, *OPN1sw2*, and *OPN1lw*) in 363 birds.

Supplementary Table 11. Contains all the homologous groups across all 363 birds, which was obtained from the Cactus alignment without a specified reference genome. This table includes all possible gene pairs within and between species and can be used to study the evolution of particular genes including all duplications. File B10K_name_map.xls allows translation of B10K sample codes, short 6 letter codes used in ortholog annotations and species latin names.

Supplementary Table 12. Contains orthologs with conserved synteny with chicken. We used the gene synteny between chicken and other species to identify the ancestral copies in the homology groups containing chicken genes. The table contains 15,671 orthologs, including one-to-one orthologs, and ancestral and novel copies from one-to-many or many-

to-many orthologs, where those copies could be distinguished. Sample codes are listed in the first row. File B10K_name_map.xls allows translation of B10K sample codes, short 6 letter codes used in ortholog annotations and species latin names.

Supplementary Table 13. Length of lineage-specific sequences for each MRCA of each order, total length of the ancestral genome of the MRCA and ratio. Orders with only one sequenced species were not included (Gaviiformes, Leptosomatiformes, Mesitornithiformes, Opisthocomiformes, Phaethontiformes, Phoenicopteriformes, and Struthioniformes).

Supplementary Table 14. List of 154 functional Passeriformes-specific genes. These gene names are assigned to the corresponding lineage-specific genes based on the Swissprot function annotation. For items with the same gene names, they were located in different regions of the ancestral genome. Species Number refers to the number of Passeriformes that were detected to contain the lineage-specific genes (out of 174 species). Average CDS length is the mean of the mapped CDS length with query bird among Passeriformes; average query CDS overlap is the mean of the ratio of mapped CDS length vs. CDS length of the query gene among Passeriformes.

Supplementary Table 15. Significance thresholds and coverage of conserved sites for expected FDR 0.05 in the different phyloP score sets.

References

1. Xu, L. *et al.* Dynamic evolutionary history and gene content of sex chromosomes across diverse songbirds. *Nat Ecol Evol* **3**, 834–844 (2019).
2. Zhang, G. *et al.* Comparative genomic data of the Avian Phylogenomics Project. *Gigascience* **3**, 26 (2014).
3. Zhang, G. *et al.* Comparative genomics reveals insights into avian genome evolution and adaptation. *Science* **346**, 1311–1320 (2014).
4. Jarvis, E. D. *et al.* Whole-genome analyses resolve early branches in the tree of life of modern birds. *Science* **346**, 1320–1331 (2014).
5. Dickinson, E. C. The Howard and Moore Complete Checklist of the Birds of the World, version 4.0 (Downloadable checklist). (2014).
6. Le Duc, D. *et al.* Kiwi genome provides insights into evolution of a nocturnal lifestyle. *Genome Biol.* **16**, 147 (2015).
7. Brown, J. W., Wang, N. & Smith, S. A. The development of scientific consensus: Analyzing conflict and concordance among avian phylogenies. *Mol. Phylogenet. Evol.*

116, 69–77 (2017).

8. Yu, G., Smith, D. K., Zhu, H., Guan, Y. & Lam, T. T.-Y. ggtree: An r package for visualization and annotation of phylogenetic trees with their covariates and other associated data. *Methods Ecol. Evol.* **8**, 28–36 (2017).
9. Luo, R. *et al.* SOAPdenovo2: An empirically improved memory-efficient short-read de novo assembler. *Gigascience* **1**, 18 (2012).
10. Gnerre, S. *et al.* High-quality draft assemblies of mammalian genomes from massively parallel sequence data. *Proc. Natl. Acad. Sci. U. S. A.* **108**, 1513–1518 (2011).
11. Li, R. *et al.* The sequence and de novo assembly of the giant panda genome. *Nature* **463**, 311–317 (2010).
12. Weisenfeld, N. I., Kumar, V., Shah, P., Church, D. M. & Jaffe, D. B. Direct determination of diploid genome sequences. *Genome Res.* **27**, 757–767 (2017).
13. Zimin, A. V. *et al.* The MaSuRCA genome assembler. *Bioinformatics* **29**, 2669–2677 (2013).
14. Kajitani, R. *et al.* Efficient de novo assembly of highly heterozygous genomes from whole-genome shotgun short reads. *Genome Res.* **24**, 1384–1395 (2014).
15. Chapman, J. A. *et al.* Meraculous: de novo genome assembly with short paired-end reads. *PLoS One* **6**, e23501 (2011).
16. Bankevich, A. *et al.* SPAdes: A new genome assembly algorithm and its applications to single-cell sequencing. *J. Comput. Biol.* **19**, 455–477 (2012).
17. Simpson, J. T. *et al.* ABySS: A parallel assembler for short read sequence data. *Genome Res.* **19**, 1117–1123 (2009).
18. English, A. C. *et al.* Mind the gap: Upgrading genomes with Pacific Biosciences RS long-read sequencing technology. *PLoS One* **7**, e47768 (2012).
19. Putnam, N. H. *et al.* Chromosome-scale shotgun assembly using an in vitro method for long-range linkage. *Genome Res.* **26**, 342–350 (2016).
20. Simão, F. A., Waterhouse, R. M., Ioannidis, P., Kriventseva, E. V. & Zdobnov, E. M. BUSCO: Assessing genome assembly and annotation completeness with single-copy

orthologs. *Bioinformatics* **31**, 3210–3212 (2015).

21. Ratnasingham, S. & Hebert, P. D. N. bold: The Barcode of Life Data System (http://www.barcodinglife.org). *Mol. Ecol. Notes* **7**, 355–364 (2007).
22. Gilbert, M. T. P. *et al.* Whole-genome shotgun sequencing of mitochondria from ancient hair shafts. *Science* **317**, 1927–1930 (2007).
23. Allentoft, M. E. *et al.* Population genomics of Bronze Age Eurasia. *Nature* **522**, 167–172 (2015).
24. Dierckxsens, N., Mardulyn, P. & Smits, G. NOVOPlasty: de novo assembly of organelle genomes from whole genome data. *Nucleic Acids Res.* **45**, e18 (2017).
25. Meng, G., Li, Y., Yang, C. & Liu, S. MitoZ: A toolkit for animal mitochondrial genome assembly, annotation and visualization. *Nucleic Acids Res.* **47**, e63 (2019).
26. Mindell, D. P., Sorenson, M. D. & Dimcheff, D. E. An extra nucleotide is not translated in mitochondrial ND3 of some birds and turtles. *Mol. Biol. Evol.* **15**, 1568–1571 (1998).
27. Katoh, K. & Standley, D. M. MAFFT multiple sequence alignment software version 7: Improvements in performance and usability. *Mol. Biol. Evol.* **30**, 772–780 (2013).
28. Jones, P. *et al.* InterProScan 5: Genome-scale protein function classification. *Bioinformatics* **30**, 1236–1240 (2014).
29. Boeckmann, B. The SWISS-PROT protein knowledgebase and its supplement TrEMBL in 2003. *Nucleic Acids Res.* **31**, 365–370 (2003).
30. Kanehisa, M. & Goto, S. KEGG: Kyoto encyclopedia of genes and genomes. *Nucleic Acids Res.* **28**, 27–30 (2000).
31. Altschul, S. F. *et al.* Gapped BLAST and PSI-BLAST: A new generation of protein database search programs. *Nucleic Acids Res.* **25**, 3389–3402 (1997).
32. She, R., Chu, J. S.-C., Wang, K., Pei, J. & Chen, N. GenBlastA: Enabling BLAST to identify homologous gene sequences. *Genome Res.* **19**, 143–149 (2009).
33. Birney, E., Clamp, M. & Durbin, R. GeneWise and Genomewise. *Genome Res.* **14**, 988–995 (2004).
34. Edgar, R. C. MUSCLE: Multiple sequence alignment with high accuracy and high

throughput. *Nucleic Acids Res.* **32**, 1792–1797 (2004).

35. Johnson, S. C. Hierarchical clustering schemes. *Psychometrika* **32**, 241–254 (1967).
36. Trapnell, C., Pachter, L. & Salzberg, S. L. TopHat: Discovering splice junctions with RNA-Seq. *Bioinformatics* **25**, 1105–1111 (2009).
37. Trapnell, C. *et al.* Transcript assembly and quantification by RNA-Seq reveals unannotated transcripts and isoform switching during cell differentiation. *Nat. Biotechnol.* **28**, 511–515 (2010).
38. Margulies, M. *et al.* Genome sequencing in microfabricated high-density picolitre reactors. *Nature* **437**, 376–380 (2005).
39. Grabherr, M. G. *et al.* Full-length transcriptome assembly from RNA-Seq data without a reference genome. *Nat. Biotechnol.* **29**, 644–652 (2011).
40. Li, W. & Godzik, A. Cd-hit: A fast program for clustering and comparing large sets of protein or nucleotide sequences. *Bioinformatics* **22**, 1658–1659 (2006).
41. Benson, G. Tandem repeats finder: A program to analyze DNA sequences. *Nucleic Acids Res.* **27**, 573–580 (1999).
42. Smit, A. F. A. and Hubley, R. and Green, P. RepeatMasker Open-4.0. (2013-2015).
43. Smit, A. F. A. & Hubley, R. RepeatModeler Open-1.0. (2010).
44. Revell, L. J. phytools: An R package for phylogenetic comparative biology (and other things). *Methods Ecol. Evol.* **3**, 217–223 (2012).
45. Faircloth, B. C. *et al.* Ultraconserved elements anchor thousands of genetic markers spanning multiple evolutionary timescales. *Syst. Biol.* **61**, 717–726 (2012).
46. Faircloth, B. C. PHYLUCE is a software package for the analysis of conserved genomic loci. *Bioinformatics* **32**, 786–788 (2016).
47. Capella-Gutiérrez, S., Silla-Martínez, J. M. & Gabaldón, T. trimAl: A tool for automated alignment trimming in large-scale phylogenetic analyses. *Bioinformatics* **25**, 1972–1973 (2009).
48. Swofford, D. L. *PAUP: Phylogenetic Analysis Using Parsimony Version 3.0, May 1990*. (Illinois Natural History Survey, 1990).

49. Kozlov, A. M., Aberer, A. J. & Stamatakis, A. ExaML version 3: A tool for phylogenomic analyses on supercomputers. *Bioinformatics* **31**, 2577–2579 (2015).
50. Sharp, P. M. & Li, W. H. An evolutionary perspective on synonymous codon usage in unicellular organisms. *J. Mol. Evol.* **24**, 28–38 (1986).
51. Jolliffe, I. T. & Greenacre, M. J. Theory and applications of correspondence analysis. *Biometrics* **42**, 223 (1986).
52. Wright, F. The ‘effective number of codons’ used in a gene. *Gene* **87**, 23–29 (1990).
53. Peden, J. F. Analysis of Codon Usage. (University of Nottingham, 1999).
54. Yuri, T., Kimball, R. T., Braun, E. L. & Braun, M. J. Duplication of accelerated evolution and growth hormone gene in passerine birds. *Mol. Biol. Evol.* **25**, 352–361 (2008).
55. Nguyen, L.-T., Schmidt, H. A., von Haeseler, A. & Minh, B. Q. IQ-TREE: A fast and effective stochastic algorithm for estimating maximum-likelihood phylogenies. *Mol. Biol. Evol.* **32**, 268–274 (2015).
56. Kalyaanamoorthy, S., Minh, B. Q., Wong, T. K. F., von Haeseler, A. & Jermiin, L. S. ModelFinder: Fast model selection for accurate phylogenetic estimates. *Nat. Methods* **14**, 587–589 (2017).
57. Hoang, D. T., Chernomor, O., von Haeseler, A., Minh, B. Q. & Vinh, L. S. UFBoot2: Improving the ultrafast bootstrap approximation. *Mol. Biol. Evol.* **35**, 518–522 (2018).
58. Mlitz, V. *et al.* Trichohyalin-like proteins have evolutionarily conserved roles in the morphogenesis of skin appendages. *J. Invest. Dermatol.* **134**, 2685–2692 (2014).
59. Mlitz, V., Hussain, T., Tschachler, E. & Eckhart, L. Filaggrin has evolved from an ‘S100 fused-type protein’ (SFTP) gene present in a common ancestor of amphibians and mammals. *Exp. Dermatol.* **26**, 955–957 (2017).
60. Rochman, M. *et al.* Profound loss of esophageal tissue differentiation in patients with eosinophilic esophagitis. *J. Allergy Clin. Immunol.* **140**, 738–749.e3 (2017).
61. Kapusta, A., Suh, A. & Feschotte, C. Dynamics of genome size evolution in birds and mammals. *PNAS* **114**, E1460–E1469 (2017).
62. Fidler, A. E., Aidala, Z., Anderson, M. G., Ortiz-Catedral, L. & Hauber, M. E.

Pseudogenisation of the short-wavelength sensitive 1 (SWS1) opsin gene in two New Zealand endemic passerine species: The Yellowhead (*Mohoua ochrocephala*) and Brown Creeper (*M. novaeseelandiae*). *Wilson J. Ornithol.* **128**, 159–163 (2016).

63. Riede, T., Suthers, R. A., Fletcher, N. H. & Blevins, W. E. Songbirds tune their vocal tract to the fundamental frequency of their song. *PNAS* **103**, 5543–5548 (2006).
