# Supplementary material for: Dense sampling of bird diversity increases power of comparative genomics
Source: Nature. 2020 Nov 11;587(7833):252–7. doi: 10.1038/s41586-020-2873-9 (PMC7759463; doi:10.1038/s41586-020-2873-9)
Supplement: Supplementary file 2 — Reporting Summary [file 41586_2020_2873_MOESM2_ESM.pdf]

nature research

Corresponding author(s): Benedict Paten, Guojie Zhang

Last updated by author(s): Jul 10, 2020

Reporting Summary

Nature Research wishes to improve the reproducibility of the work that we publish. This form provides structure for consistency and transparency in reporting. For further information on Nature Research policies, see Authors & Referees and the Editorial Policy Checklist .

Statistics

For all statistical analyses, confirm that the following items are present in the figure legend, table legend, main text, or Methods section.

n/a	Confirmed	
☐	☒	The exact sample size (*n*) for each experimental group/condition, given as a discrete number and unit of measurement
☐	☒	A statement on whether measurements were taken from distinct samples or whether the same sample was measured repeatedly
☐	☒	The statistical test(s) used AND whether they are one- or two-sided<br>*Only common tests should be described solely by name; describe more complex techniques in the Methods section.*
☒	☐	A description of all covariates tested
☐	☒	A description of any assumptions or corrections, such as tests of normality and adjustment for multiple comparisons
☐	☒	A full description of the statistical parameters including central tendency (e.g. means) or other basic estimates (e.g. regression coefficient) AND variation (e.g. standard deviation) or associated estimates of uncertainty (e.g. confidence intervals)
☐	☒	For null hypothesis testing, the test statistic (e.g. *F*, *t*, *r*) with confidence intervals, effect sizes, degrees of freedom and *P* value noted<br>*Give P values as exact values whenever suitable.*
☒	☐	For Bayesian analysis, information on the choice of priors and Markov chain Monte Carlo settings
☒	☐	For hierarchical and complex designs, identification of the appropriate level for tests and full reporting of outcomes
☐	☒	Estimates of effect sizes (e.g. Cohen's *d*, Pearson's *r*), indicating how they were calculated

Our web collection on statistics for biologists contains articles on many of the points above.

Software and code

Policy information about availability of computer code

Data collection	No software was used.
Data analysis	Common bioinformatic and statistical analysis software packages were used, including: ggtree (v2.2.1), SOAPdenovo (v2.04, version Jun-2015, version Jul-2012), Gapcloser (v1.12), Allpaths-LG (v49184, v50081, v50191, v52117, v52485, v52488, version Jul-2013, version Dec-2016), SuperNova (v1.0, v1.1, v1.2), MaSuRCA (v3.1.1, v3.2.1), Platanus (v.1.2.1, v1.2.4), Meraculous (v2.0.4), Spades (v3.5.0), Abyss (v.1.9.0), PBJelly (v15.8.24), Hi-Rise (version July2015), BUSCO (v3), BLAST (v2.2.26), tblastn (v2.2.2), genBlastA (v1.0.4), GeneWise (wise2.4.1), MUSCLE (v3.8.31), TopHat (v2.1.1), Cufflinks (v2.2.1), Newbler (v2.9), Trinity (trinityrnaseq_r20140717), cd-hit (v4.6.6), InterPro (v5.24-63.0), SwissProt (release-2018_07), KEGG (v81), Tandem Repeats Finder (v4.07b), RepeatMasker (v4.0.7), RepBase (v20170127), RepeatModeler (v1-0-8), phytools (v0.7-20), PHYLUCE (commit 69e7849), mafft (v7.4, v7.313), TrimAl (v1.4.rev15), PAUP (v4a164), ExaML (v3.0.9), Cactus (commit f88f23d), Blastp (v2.2.26), CodonW (v1.4.2), NOVOPlasty (v2.7.2), MitoZ (v2.3), IQ-TREE (v1.6), phyloFit (v 1.5), phyloP (v 1.5), PHAST (v 1.5), HAL toolkit (v2.1), Wiggletools (v1.2.3) and NONCODE (v5). Specific parameters used during run-time are provided in Supplementary File 1 when appropriate. Custom scripts are open source and available on GitHub page https://github.com/B10KGenomes/annotation .

For manuscripts utilizing custom algorithms or software that are central to the research but not yet described in published literature, software must be made available to editors/reviewers. We strongly encourage code deposition in a community repository (e.g. GitHub). See the Nature Research guidelines for submitting code & software for further information.

Data

Policy information about availability of data

All manuscripts must include a data availability statement. This statement should provide the following information, where applicable:

- Accession codes, unique identifiers, or web links for publicly available datasets
- A list of figures that have associated raw data
- A description of any restrictions on data availability

Genome sequencing data, the genome assemblies and annotations of 267 species generated in this study have been deposited in the NCBI SRA and GenBank under PRJNA545868 (https://www.ncbi.nlm.nih.gov/bioproject/PRJNA545868). The above data have also been deposited in the CNSA of CNGBdb with accession number CNP0000505 (https://db.cngb.org/search/project/CNP0000505/). The mitochondrial genomes and annotations of 336 species have been deposited in the NCBI GenBank under PRJNA545868. Data generated and analysed during this study are included in the supplementary information files or Mendeley Data under reserved DOI doi:10.17632/fnpwzj37gw. The whole genome alignment of the 363 birds is available at https://alignment-output.s3.amazonaws.com/birds-final.hal. We have created a UCSC browser hub for all 363 species, available by placing the hub URL https://comparative-genomics-hubs.s3-us-west-2.amazonaws.com/b10k_hub.txt into the "My Hubs" tab of the "Track Hubs" section.

Field-specific reporting

Please select the one below that is the best fit for your research. If you are not sure, read the appropriate sections before making your selection.

[x] Life sciences   [ ] Behavioural & social sciences   [ ] Ecological, evolutionary & environmental sciences

For a reference copy of the document with all sections, see nature.com/documents/nr-reporting-summary-flat.pdf

Life sciences study design

All studies must disclose on these points even when the disclosure is negative.

Sample size	A determination of sample size as performed for experimental studies was not applicable in this study. Preserved tissue samples for genome sequencing were selected to represent all bird families based on the taxonomic system. One target bird species has one preserved tissue sample used for the DNA extraction. For Rissa tridactyla (OUT-0021), two individual samples are used, because a single preserved tissue sample cannot meet the amount of DNA required for sequencing.
Data exclusions	We excluded genome assemblies that had low assembly quality or were potentially contaminated. 236 genomes remained from a total 272 sequenced species after excluding the following samples:<br>- 13 genomes were removed due to poor genome assembly quality (scaffold N50 <10 kb and/or total assembly length <0.9 Gb);<br>- 13 genomes were removed because of potential contamination of the sample<br>- 10 genomes were redundant with a genome of better quality available on NCBI or from external labs.
Replication	Replication was not applicable because no experimentation was performed in this study.
Randomization	Randomization was not applicable because no experimentation was performed in this study.
Blinding	Blinding was not applicable because no experimentation was performed in this study.

Reporting for specific materials, systems and methods

We require information from authors about some types of materials, experimental systems and methods used in many studies. Here, indicate whether each material, system or method listed is relevant to your study. If you are not sure if a list item applies to your research, read the appropriate section before selecting a response.

Materials & experimental systems

n/a	Involved in the study	
[x]	[ ]	Antibodies
[x]	[ ]	Eukaryotic cell lines
[x]	[ ]	Palaeontology
[ ]	[x]	Animals and other organisms
[x]	[ ]	Human research participants
[x]	[ ]	Clinical data

Methods

n/a	Involved in the study	
[x]	[ ]	ChIP-seq
[x]	[ ]	Flow cytometry
[x]	[ ]	MRI-based neuroimaging

Animals and other organisms

Policy information about studies involving animals; ARRIVE guidelines recommended for reporting animal research

Laboratory animals	Study did not involve laboratory animals.
Wild animals	Study did not involve wild animals.
Field-collected samples	Study did not collect new samples in the field and did not perform in vivo animal research as defined in the ARRIVE guidelines. The study used preserved tissue samples sourced from museums and other natural history collections. The institutions, the field collectors, curators and staff at the relevant museums and natural history collections are listed in Supplementary Table S1.
Ethics oversight	No ethical approval was required for the study because no live samples were collected and no live experimentation was performed. Museums listed in Supplementary Table 1 issued written permission to sequence, analyze, and publish the genetic material provided by them to the B10K consortium.

Note that full information on the approval of the study protocol must also be provided in the manuscript.
